# Safety Profile of L-Arginine Infusion in Moderately Severe Falciparum Malaria

**DOI:** 10.1371/journal.pone.0002347

**Published:** 2008-06-11

**Authors:** Tsin W. Yeo, Daniel A. Lampah, Retno Gitawati, Emiliana Tjitra, Enny Kenangalem, Donald L. Granger, J. Brice Weinberg, Bert K. Lopansri, Ric N. Price, David S. Celermajer, Stephen B. Duffull, Nicholas M. Anstey

**Affiliations:** 1 International Health Division, Menzies School of Health Research, Charles Darwin University, Darwin, Northern Territory, Australia; 2 Menzies School of Health Research-National Institute of Health Research and Development Research Program, and District Ministry of Health, Timika, Papua, Indonesia; 3 National Institute of Health Research and Development, Jakarta, Indonesia; 4 Division of Infectious Diseases, VA Medical Centers, University of Utah, Salt Lake City, Utah, United States of America; 5 Division of Hematology-Oncology, VA Medical Centers, Duke University School of Medicine, Durham, North Carolina, United States of America; 6 Division of Medicine, Royal Darwin Hospital, Darwin, Northern Territory, Australia; 7 Centre for Vaccinology and Tropical Medicine, Nuffield Department of Clinical Medicine, John Radcliffe Hospital, Oxford, United Kingdom; 8 Department of Medicine, University of Sydney and Department of Cardiology, Royal Prince Alfred Hospital, Sydney, Australia; 9 School of Pharmacy, University of Otago, Dunedin, New Zealand; Walter and Eliza Hall Institute of Medical Research, Australia

## Abstract

**Background:**

L-arginine infusion improves endothelial function in malaria but its safety profile has not been described in detail. We assessed clinical symptoms, hemodynamic status and biochemical parameters before and after a single L-arginine infusion in adults with moderately severe malaria.

**Methodology and Findings:**

In an ascending dose study, adjunctive intravenous L-arginine hydrochloride was infused over 30 minutes in doses of 3 g, 6 g and 12 g to three separate groups of 10 adults hospitalized with moderately severe *Plasmodium falciparum* malaria in addition to standard quinine therapy. Symptoms, vital signs and selected biochemical measurements were assessed before, during, and for 24 hours after infusion. No new or worsening symptoms developed apart from mild discomfort at the intravenous cannula site in two patients. There was a dose-response relationship between increasing mg/kg dose and the maximum decrease in systolic (ρ = 0.463; Spearman's, p = 0.02) and diastolic blood pressure (r = 0.42; Pearson's, p = 0.02), and with the maximum increment in blood potassium (r = 0.70, p<0.001) and maximum decrement in bicarbonate concentrations (r = 0.53, p = 0.003) and pH (r = 0.48, p = 0.007). At the highest dose (12 g), changes in blood pressure and electrolytes were not clinically significant, with a mean maximum decrease in mean arterial blood pressure of 6 mmHg (range: 0–11; p<0.001), mean maximal increase in potassium of 0.5 mmol/L (range 0.2–0.7 mmol/L; p<0.001), and mean maximal decrease in bicarbonate of 3 mEq/L (range 1–7; p<0.01) without a significant change in pH. There was no significant dose-response relationship with blood phosphate, lactate, anion gap and glucose concentrations. All patients had an uncomplicated clinical recovery.

**Conclusions/Significance:**

Infusion of up to 12g of intravenous L-arginine hydrochloride over 30 minutes is well tolerated in adults with moderately severe malaria, with no clinically important changes in hemodynamic or biochemical status. Trials of adjunctive L-arginine can be extended to phase 2 studies in severe malaria.

**Trial Registration:**

ClinicalTrials.gov NCT00147368

## Introduction

Although treatment with the rapidly acting anti-parasitic drug, artesunate has significantly improved survival in severe malaria, the case-fatality rate remains high [1]. Adjunctive treatments that target underlying pathophysiologic processes in severe malaria may reduce mortality further but none have been shown to be efficacious to date [2], [3].

L-arginine is the substrate for synthesis of nitric oxide (NO) from NO synthase, and has been proposed as a potential adjunctive therapy in severe malaria [4], [5]. The rationale for this is based on previous findings in severe malaria of impaired NO production [5], [6], hypoargininemia [4], near-universal impairment of NO-dependent endothelial function [5] and a close association between the improvement in endothelial function and recovery of plasma L-arginine concentrations after treatment of severe malaria [7]. NO down-regulates endothelial inflammation [8] and reduces the cytoadherence of parasitized erythrocytes *in vitro* [9]. Endothelial dysfunction, a measure of both impaired endothelial cell NO bioavailability and endothelial cell activation [10], may exacerbate other underlying processes in severe malaria including cytoadherence of parasitised red cells to activated endothelial cells, microvascular obstruction and tissue hypoxia. In severe malaria, impaired endothelial function is associated with markers of impaired perfusion, endothelial activation, and with increased parasite biomass [5]. We have recently demonstrated that L-arginine infusion is able to improve NO bioavailability and endothelial function in patients with moderately severe malaria [5], suggesting the potential for a similar effect if used as adjunctive therapy in severe malaria.

Intravenous L-arginine has been used for over 40 years in routine clinical practice to assess the integrity and function of the hypothalamic-pituitary axis [11], where it is generally well-tolerated when infused in large doses (30g; ∼0.5 g/kg) over 30 minutes. More recently it has also been evaluated as a therapeutic agent to improve endothelial function in cardiovascular diseases. Studies in healthy volunteers [12] and patients with cardiovascular disease [13], [14] have shown L-arginine infusion to be safe with minimal side effects. Adverse events reported with intravenous L-arginine in these settings include modest dose-dependent effects on blood pressure, potassium and phosphate [15], [16] and a potential for effects on acid base status [14] and blood glucose [17], [18] . However, data on the hemodynamic and biochemical changes resulting from infusion of L-arginine in patients with acute inflammatory states have not been well documented.

As a prelude to assessing the safety and potential utility of adjunctive L-arginine infusion in severe malaria, we undertook a safety, pharmacokinetic and efficacy study in patients with moderately severe falciparum malaria (MSM). The efficacy of L-arginine in improving endothelial function and NO production in these patients has been reported previously [5]. Here we describe detailed data on the safety of L-arginine infusion in the same group of patients, with a focus on the hemodynamic and biochemical changes and other potential adverse effects previously reported or hypothesized in other clinical settings [14]. In seeking to evaluate clinical safety we specifically sought the maximum change in the vital signs and biochemical markers at any time during and after L-arginine infusion compared to baseline readings. While not reflecting the mean changes across the infusion and post-infusion periods, this approach identified the “worst-case scenario”, however transient, for each parameter.

## Methods

The protocol for this trial is available as supporting information; see Protocol S1.

### Study Site and Study design

The study was conducted at Mitra Masyarakat Hospital (RSMM) in Timika, Papua, Indonesia, a region with unstable transmission of multidrug-resistant *Plasmodium falciparum* and *P. vivax* [19]. The design was a single ascending dose study of the safety, pharmacokinetics and preliminary efficacy of adjunctive L-arginine infusion. Patients were sequentially enrolled after written informed consent was obtained.

### Participants

Patients comprised adults ≥18 years of age with moderately severe falciparum malaria (MSM) defined as fever or history of fever in the past 48 hours, with >1000 asexual *P. falciparum* parasites/µL (a threshold for clinical falciparum malaria in Papua [20]), with no other etiology identified, requiring inpatient parenteral therapy because of inability to tolerate oral therapy. Patients exhibiting WHO warning signs or criteria for severe malaria were excluded [21]. Other exclusion criteria included: pregnant or breastfeeding women, patients treated with anti-malarials for >18 hours, mixed *P. falciparum/P. vivax infections,* diabetes, known cardiac, renal or hepatic disease, concurrent infection and a haemoglobin <6 g/dl, systolic blood pressure <100 mmHg, a baseline venous bicarbonate level <20 mEq/L, potassium ≥4.2 mmol/L, glucose <4 mmol/L, or chloride >106 mmol/L. Individuals were treated with intravenous quinine in accordance with national guidelines and also received doxycycline or clindamycin. Decisions regarding provision of additional supportive care, including antibiotics and fluid administration, were made by the treating clinician.

### L-arginine Infusion

L-arginine hydrochloride (Pharmalab, Sydney, Australia) was diluted in 100 ml normal saline (or sufficient to provide a concentration ≤10% w/v) and administered intravenously by an infusion pump over 30 minutes at doses of 3 g, 6 g and 12 g. Intravenous quinine was continued during infusion of L-arginine, through a separate cannula.

### Clinical Observations

Upon enrolment and before infusion, patients had a standardized clinical history and physical examination recorded. During infusion patients were monitored with serial assessments of the following symptoms (nausea, vomiting, fever, giddiness, numbness of hands or feet, flushing of face, difficulty breathing, cough, pain at infusion site, rash, WHO dangers signs [21] and vital signs (systolic and diastolic blood pressure, pulse rate, respiratory rate, temperature) at 10 minute intervals. After infusion, vital signs and symptoms were obtained at intervals of 15 minutes for the first hour, 30 minute for the next 3 hours, 4 hourly until 24 hours, and 12 hourly until discharge. In total, 21 observations were obtained, 4 before, 3 during and 14 after infusion. Before, during and up to four hours after infusion, patients were also monitored for arrhythmias with a continuous electrocardiogram attached to the limb leads. We used an automated sphygmomanometer to measure blood pressure and a digital thermometer for axillary temperature.

### Biochemical Measurements

Venous blood samples were taken 30 minutes before and immediately prior to the start of the infusion and again at the end of the 30 minute infusion. Further samples were taken 5, 20, 45, 60, 90 minutes and 2, 4, 8, 24 hours after the end of the infusion. In total, 12 venous blood samples were taken, 2 before infusion and 10 after. Measurements for blood potassium, glucose, bicarbonate, pH, chloride, anion gap and phosphate concentrations were performed on all samples. These were chosen based on previously reported or hypothesized effects of L-arginine on these markers. Lactate concentrations were measured on the venous sample obtained immediately prior to infusion and at 4 hours. Plasma creatinine, creatine kinase, bilirubin and liver enzymes (AST, ALT and alkaline phosphatase) were measured at baseline and 24 hours after infusion.

### Laboratory Methods

Electrolytes, glucose, acid-base parameters and were measured using a bedside biochemical analyser (i-STAT Corp, Windsor, NJ, USA). Plasma phosphate, serum creatinine, bilirubin, liver enzymes and creatine kinase were measured using colorimetric and enzymatic methods (Cobas 800, Roche Diagnostics).

### Statistical Methods

Mean values of the vital sign and biochemical parameters before infusion were compared to mean values obtained immediately after infusion using a paired t-test (for continuous variables with a normal distribution or variables log transformed to a normal distribution). If parameters were not normally distributed, values before and after infusion were compared by the Wilcoxon Signed Rank Test. Because analysis in this manner could mask transient but potentially clinically significant changes in a minority of subjects, we also analyzed the maximum change in the vital signs and biochemical markers at any time during and after L-arginine infusion compared to baseline readings. This gave a “worst-case scenario”, however transient, for each parameter. The mean of the pre-infusion values in each patient was subtracted from the lowest (SBP, DBP, glucose, bicarbonate, phosphate, pH) and highest (pulse rate, respiratory rate, potassium, chloride and anion gap) measurement during infusion or in the two hours following infusion and expressed as mean maximum change. Relationships between the maximum changes and the dose weight ratio (expressed as mg/kg) were assessed by Pearson's correlation matrix (r), or Spearman's rank correlation coefficient (ρ) if the dependent variable was not normally distributed. A general linear model was used to assess dose-response relationships. All analyses were performed with Stata 9.2 software (Statacorp). A two sided value of p<0.05 was considered significant.

Ethical approval was obtained from the respective committees of the National Institute of Health Research and Development, Jakarta, Indonesia and the Menzies School of Health Research, Darwin, Australia and the trial was registered in clinicaltrials.gov as NCT00147368. The funders had no role in the study design, data collection, analysis, decision to publish or preparation of the manuscript.

## Results

### Patients

A total of 30 patients received a single L-arginine infusion intravenously, at doses of 3 g (n = 10), 6 g (n = 10) and 12 g (n = 10). The corresponding mean mg/kg doses in each group were 53 mg/kg (range: 45–63), 105 mg/kg (range: 87–140) and 205 mg/kg (range: 155–240), respectively. **Table 1** shows baseline patient characteristics. The mean time to enrolment was 6 hours after starting intravenous quinine (range 2–12 hours).

**Table 1:** Baseline characteristics of patients who received L-arginine infusions

Number	30
Age; mean (range), y	28 (18–54)
Males, No. (%)	20 (67)
Weight; mean (range), kg	58 (42–70)
Ethnicity, No. (%) Papuan highlander	23 (77)
Systolic blood pressure; mean (range), mmHg	109 (90–138)
Mean arterial blood pressure; mean (range), mmHg	83 (66–116)
Percentage hypertensive on enrolment	0%
Pulse rate; mean (range), beats/minute	80 (54–116)
Respiratory rate; mean (range), breaths/minute	24 (18–32)
Temperature; mean (range), °C	37 (34.8–40.2)
Potassium; mean (range), meq/L	3.2 (2.6–3.8)
Bicarbonate; mean (range), meq/L	24.1 (20.1–29.6)
Chloride; mean (range), meq/L	102 (95–105)
Phosphate; mean (range), meq/L	1.1 (0.7–2.2)
Glucose; mean (range), mmol/L	7.6 (4.8–14.5)
Haemoglobin; mean (95% CI), g/L	123 (75–170)
Plasma L-arginine; mean (95%CI), µmol/L	37 (33–43)
Lactate concentration; mean (95%CI), mmol/L	1.5 (1.2–1.8)
Parasite density; geometric mean (range), µl^−1^	17,221 (890–281,864)

### Clinical adverse effects during L-arginine infusion

Two patients, one receiving 3 g and another 12 g developed mild discomfort over the intravenous cannula site during infusion, but no signs of phlebitis were evident and no extravasations occurred. No other patients developed new symptoms. There was also no worsening of existing symptoms either during or after infusion of L-arginine; in particular, no new or worsening complaints of headaches, vomiting, nausea, flushing, giddiness, dyspnea, cough, diarrhea, lethargy and anorexia. No allergic reactions were seen.

### Effect of L-arginine on vital signs

**Figure 1:**
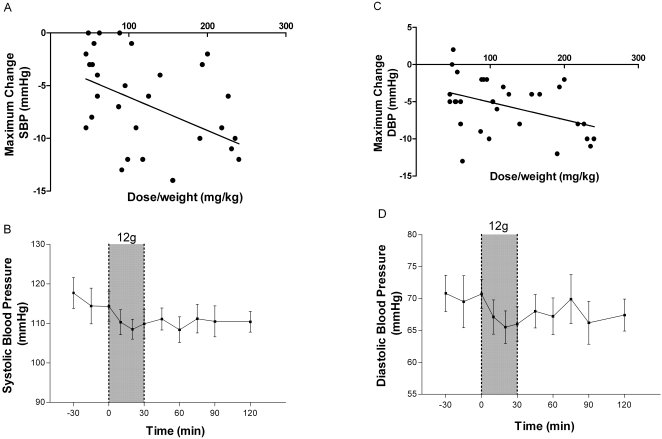
Effects of L-arginine hydrochloride infusion on blood pressure. Figure 1A: Relationship between dose/weight (mg/kg) of infused L-arginine and the maximal fall in systolic blood pressure at any time during or in the two hours following infusion (n = 30; ρ = −0.463, p = 0.015). Figure 1B: The systolic blood pressure profiles of patients before, during and up to 120 minutes after 30 minute administration of the highest dose of L-arginine (12 g; n = 10). Shaded area indicates time of L-arginine infusion. Dots and bars indicate mean±SEM. Figure 1C: Relationship between dose/weight (mg/kg) of infused L-arginine and the maximal fall in diastolic blood pressure at any time during or in the two hours following infusion (n = 30; r = −0.42, p = 0.02). Figure 1D: The diastolic blood pressure profiles of patients before, during and up to 120 minutes after 30 minute administration of the highest dose of L-arginine (12 g; n = 10). Shaded area indicates time of L-arginine infusion. Dots and bars indicate mean±SEM.

There were no statistically or clinically significant effects on pulse rate (data not shown) or respiratory rate (data not shown) before and during or after infusion of any dose of L-arginine, with continuous electrocardiography remaining normal throughout. Although the mean systolic and diastolic blood pressure did not change significantly following the 3 g and 6 g arginine infusion, patients given 12 g had a transient mean decrease in systolic (5 mmHg [95%CI 3 to 8 mmHg]; p = 0.01) and diastolic (5 mmHg [95%CI 0.5 to 9 mmHg]; p = 0.03) blood pressures measured immediately at the end of infusion which returned towards baseline within 15 minutes (**Figure 1B and 1D**). The *maximum* falls in blood pressure at any time during or in the two hours after infusion were also assessed. The maximum decrease in both the systolic (ρ = 0.463; Spearman's, p = 0.002; **Figure 1A** **)** and diastolic blood pressure (r = 0.42; Pearson's, p = 0.02; **Figure** **1C** **)** correlated with increasing mg per kg dose. Each increment of dose was associated with an increase in the mean maximum decrement in systolic blood pressure of 3 mmHg (p = 0.004; 95%CI: 1–5 mmHg) but not in diastolic blood pressure (p = 0.094). Patients who received 3, 6 and 12 g had mean maximum decreases in mean arterial blood pressure of 1.2 mmHg (range 0 to 8), 4 mmHg (range 1 to 10) and 6 mmHg (range: 0 to 11; p<0.001) respectively. In no patient did systolic blood pressure fall to 90 mmHg or less. Table 2 shows the maximum changes in the systolic and diastolic blood pressures up to 2 hours after the start of infusion when compared to pre-infusion values in each dosing group.

**Table 2:** Maximum changes in parameters at any time during and for 2 hours after L-arginine infusion (3–12g) compared to pre infusion values (mean maximum change [range])

Dose	3g	6g	12g
SBP (mmHg)	−3 (0 to −8)	−6 (0 to −12)	−9 (−2 to −18)
DBP (mmHg)	−4 (+2 to −10)	−5 (−2 to −8)	−6 (−2 to −10)
Potassium (mmol/L)	0.2 (−0.1 to 0.2)	0.3 (0.1 to 0.4)	0.5 (0.2 to 0.7)
HCO3 (meq/L)	−1 (1 to −3)	−1.5 (1 to −3.5)	−3 (−1 to −6)
pH	−0.005 (−0.001 to −0.05)	−0.01 (+0.05 to −0.05)	−0.04 (−0.02 to −0.06)
Chloride (mmol/L)	2 (0 to 3)	2 (−1 to 3)	3 (2 to 5)
Lactate (mmol/L)	−0.05 (1 to −2)	−0.34 (0.05 to −2)	−0.34 (1 to −2)
Anion Gap	0 (−4 to 2)	−1 (−5 to 1)	−2 (−2 to 3)
Phosphate (mg/dL)	0.1 (−0.1 to 0.2)	0.1 (−0.2 to 0.2)	0.1 (−0.1 to 0.2)
Glucose (mmol/L)	−1.1 (−3.1 to 0.3)	−0.94 (−3.1 to 0.47)	−0.64 (−1.3 to 1.3)

### Effect of L-arginine on electrolytes, pH and glucose

**Figure 2:**
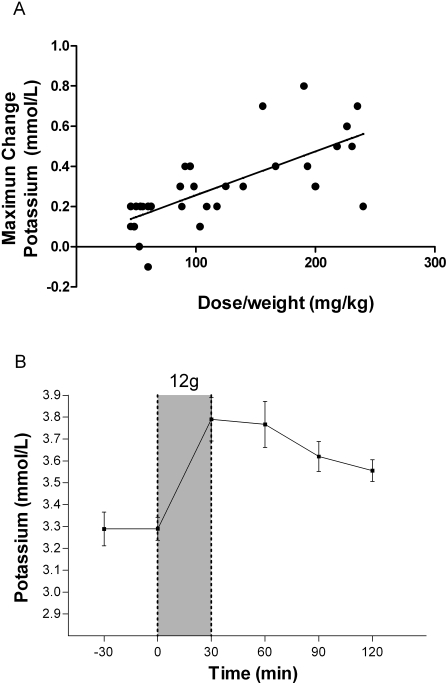
Effects of L-arginine hydrochloride infusion on whole blood potassium concentrations. Figure 2A: Relationship between dose/weight (mg/kg) of infused L-arginine and the maximal increase in whole blood potassium concentration at any time during or in the two hours following infusion (n = 30; r = 0.73, p<0.001). Figure 2B: Whole blood potassium concentrations of patients before and after 30 minute administration of the highest dose of L-arginine (12 g; n = 10). Shaded area indicates time of L-arginine infusion. Dots and bars indicate mean±SEM.

**Figure 3:**
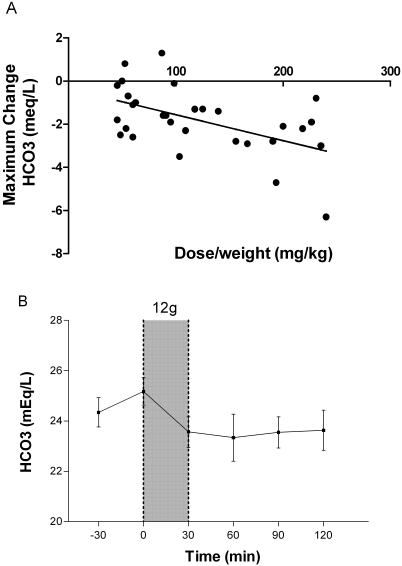
Effects of L-arginine hydrochloride infusion on venous blood bicarbonate concentrations. Figure 3A: Relationship between dose/weight (mg/kg) of infused L-arginine and the maximal decrease in venous blood bicarbonate concentration at any time during or in the two hours following infusion (n = 30; r = −0.53, p = 0.003). Figure 3B: Venous blood bicarbonate concentrations of patients before and after 30 minute administration of the highest dose of L-arginine (12 g; n = 10). Shaded area indicates time of L-arginine infusion. Dots and bars indicate mean±SEM.

**Figure 4:**
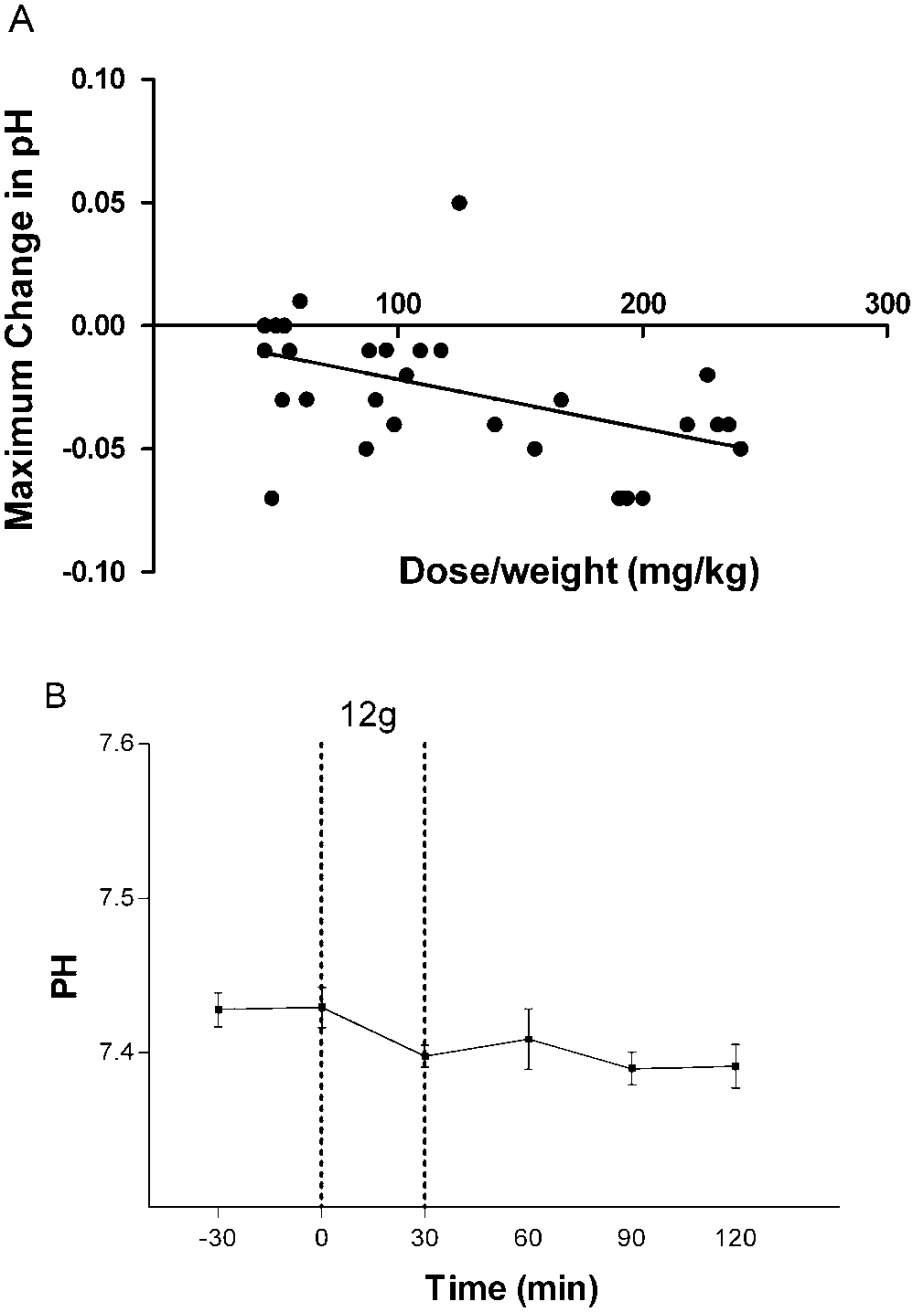
Effects of L-arginine hydrochloride infusion on venous blood pH. Figure 4A: Relationship between dose/weight (mg/kg) of infused L-arginine and the maximal decrease in venous blood pH at any time during or in the two hours following infusion (n = 30; r = −0.48, p = 0.007). Figure 4B: Venous blood pH in patients before and after 30 minute administration of the highest dose of L-arginine (12 g; n = 10). Shaded area indicates time of L-arginine infusion. Dots and bars indicate mean±SEM.

**Figure 5:**
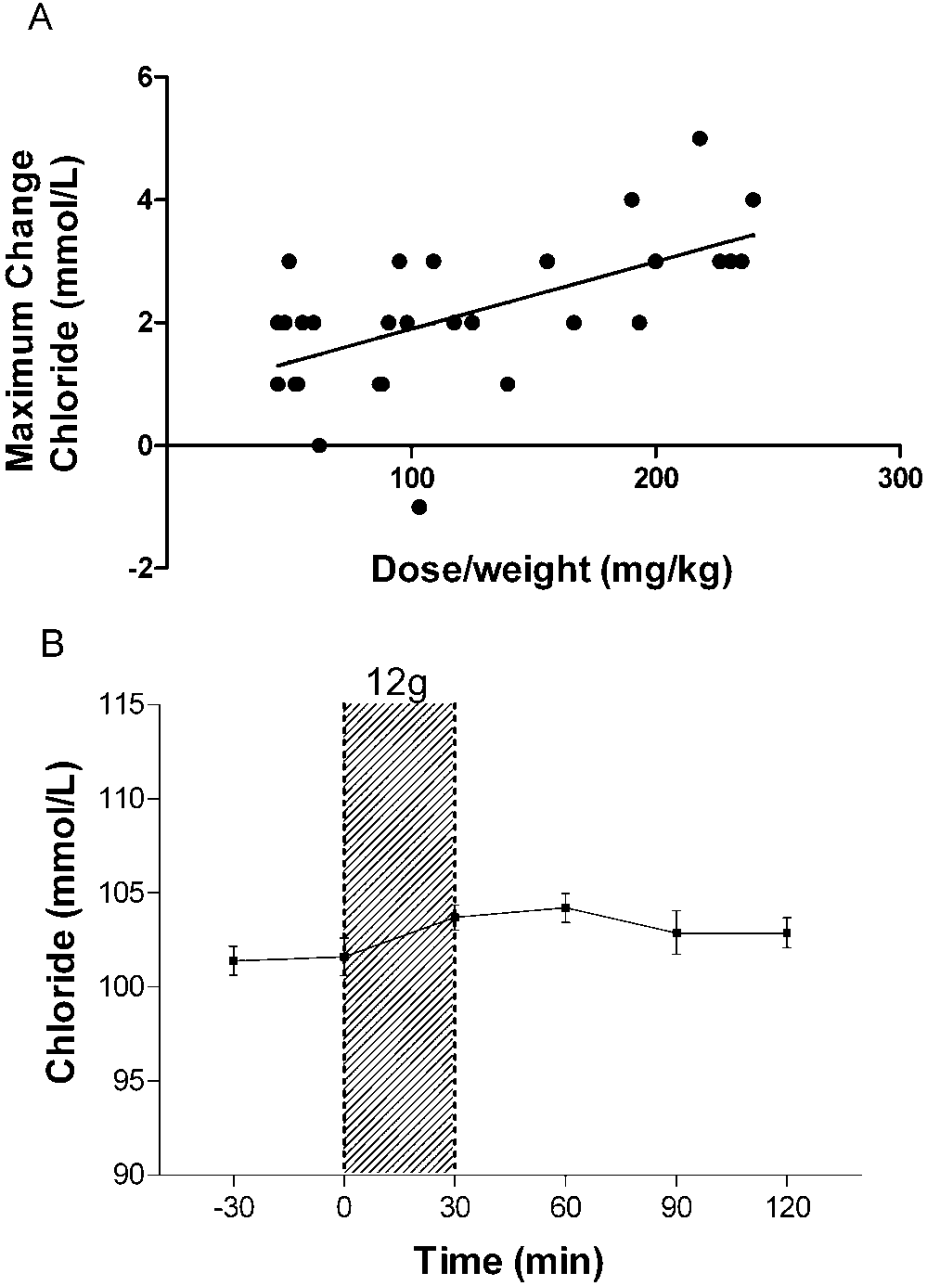
Effects of L-arginine hydrochloride infusion on venous blood chloride concentrations. Figure 5A: Relationship between dose/weight (mg/kg) of infused L-arginine and the maximal decrease in venous blood chloride at any time during or in the two hours following infusion (n = 30, r = 0.6, p<0.001). Figure 5B: Venous blood chloride in patients before and after 30 minute administration of the highest dose of L-arginine (12 g; n = 10). Shaded area indicates time of L-arginine infusion. Dots and bars indicate mean±SEM

The maximum increases in potassium, chloride and anion gap, and the maximum decreases in bicarbonate, pH, glucose, and phosphate were assessed following L-arginine infusion. Each dose increase of 100 mg per kg of L-arginine was associated with a mean maximum increment in blood potassium of 0.2 mmol/L(95%CI: 0.1–0.3)[r = 0.7; Pearson's, p<0.001; **Figure 2A**]. In patients given 12 g there was a statistically significant mean increase in potassium of 0.5 mmol/L (range 0.2 to 0.7 mmol/L; p<0.001; **Figure 2B**), peaking at the completion of infusion. In no patient did potassium concentrations rise above 4.6 mmol/L (normal range: 3.3–5.0 mmol/L). For bicarbonate concentrations, a maximum decrement in bicarbonate concentrations of 1.2 meq/L (95%CI: 1.9–0.45)(r = 0.53; Pearson's, p = 0.003; **Figure 3A**) was noted with each increase of 100 mg/kg L-arginine; in those receiving the 12 g dose, the mean decrease in bicarbonate was 3 mEq/L (range 1 to 7; p<0.01; **Figure 3B**). There was a also a mean maximal decrease in pH of 0.02 (95%CI: 0.03–0.005)(r = 0.48; Pearson's, p = 0.007**;** **Figure 4A**) per 100 mg/kg increase in L-arginine dose, but there was no significant change in mean pH in the patients given 12 g (**Figure 4B**). No patient had a bicarbonate concentration which dropped below 17 mEq/L (normal range 20–30 mEq/L) or a pH that fell below 7.34. There was a mean increment in chloride of 1.0 mmol/L(95%CI: 0.5–1.6; r = 0.6; Pearson's' p<0.001) per 100 mg/kg increase in L-arginine dose (**Figure 5A** **)**. Following the 12 g dose, mean chloride concentrations increased from 101 (95%CI: 99 to 102) to 103 (95%CI: 102 to 104) mmol/L (normal range 97–110 mmol/L) **(** **Figure 5B** **)**, with a mean maximal increase of 3 mmol/L (range 1 to 5; p<0.001).

When categorized by dosage group (3g vs 6g vs 12g), there was a linear-dose response, with each dose increment associated with an increase in the mean maximum potassium increment (0.19 mmol/L [95%CI: 0.12 to 0.25 mmol/L] p<0.001), the mean maximum bicarbonate decrement (1.9 mEq/L [95%CI: 0.12 to 4.8 mEq/L]; p = 0.004) and the mean maximum pH decrement (0.017 [95%CI: 0.006 to 0.027]; p = 0.003) **(** **Table 2** **)**.

**Figure 6:**
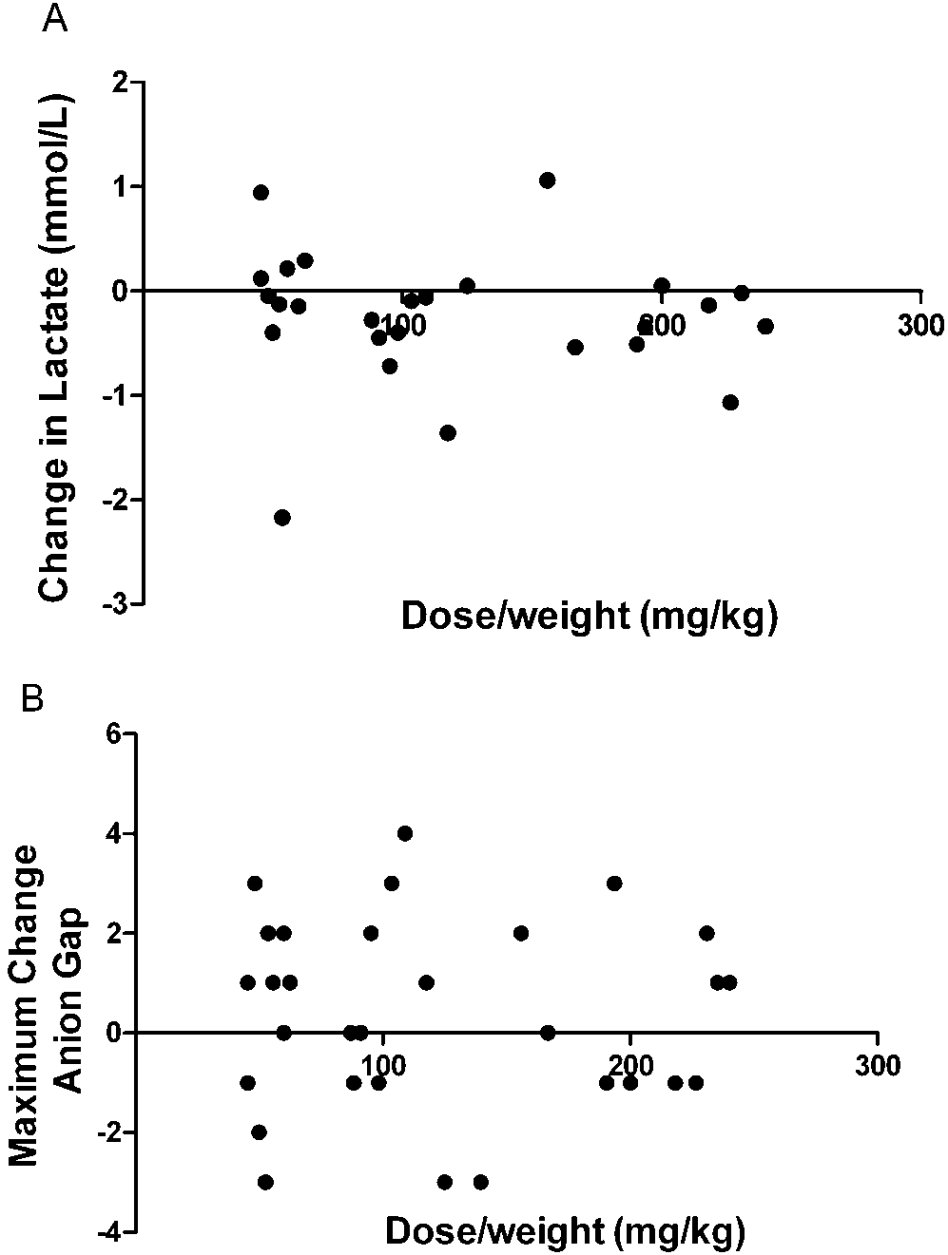
Effects of L-arginine hydrochloride infusion on venous blood lactate concentrations and anion gap. Figure 6A: Relationship between dose/weight (mg/kg) of L-arginine and change in venous blood lactate before and 4 hours after infusion (n = 30; ns). Figure 6B: Relationship between dose/weight (mg/kg) of infused L-arginine and the maximal change in venous anion gap at any time during or in the two hours following infusion (n = 30; ns).

**Figure 7:**
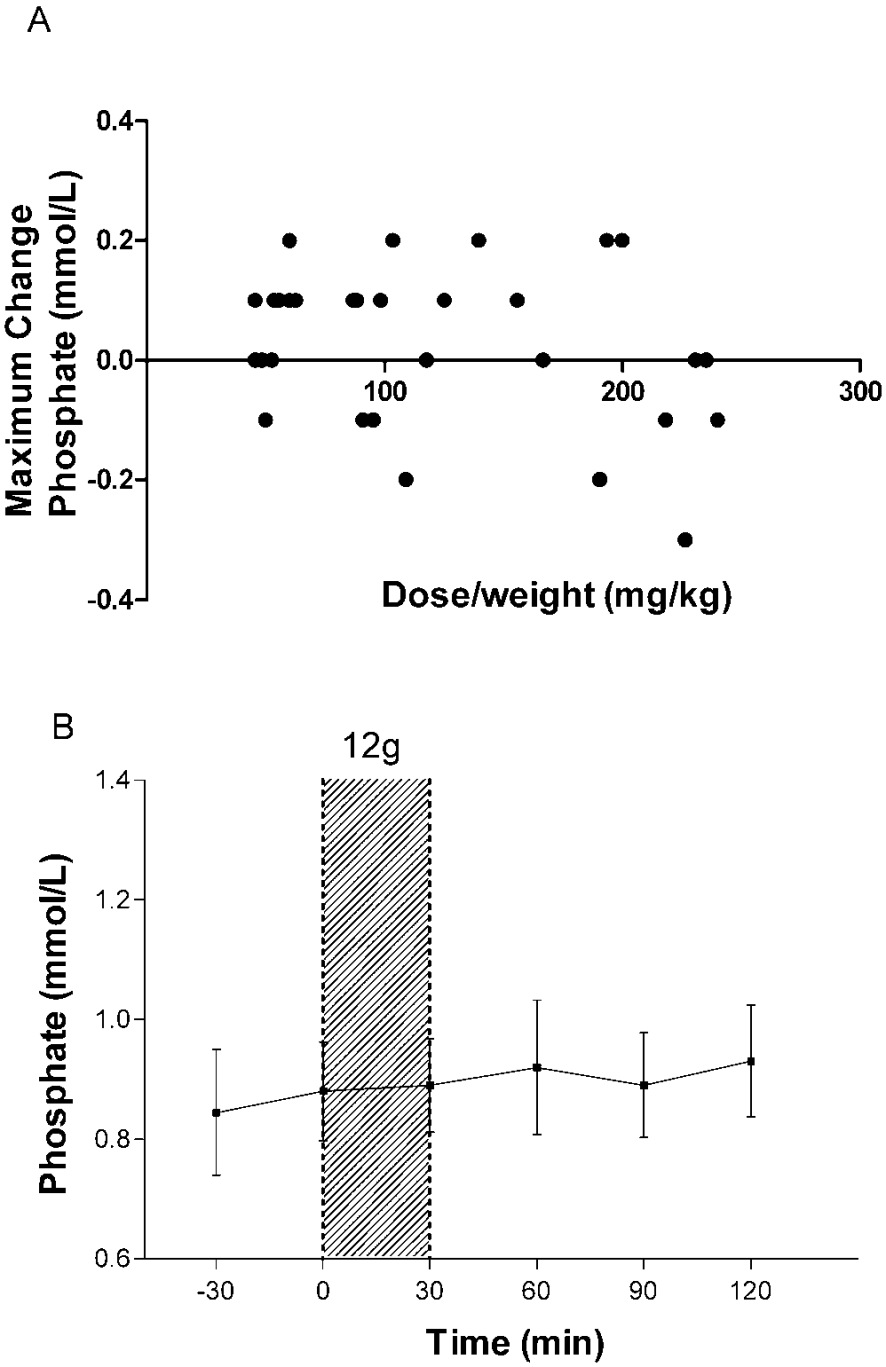
Effects of L-arginine hydrochloride infusion on plasma phosphate concentrations. Figure 7A: Relationship between dose/weight (mg/kg) of infused L-arginine and the maximal decrease in plasma phosphate concentration at any time during or in the two hours following infusion (n = 30; r = −0.06, p = 0.8). Figure 7B: Plasma phosphate concentrations of patients before and after 30 minute administration of the highest dose of L-arginine (12 g; n = 10).. Shaded area indicates time of L-arginine infusion. Dots and bars indicate mean±SEM.

**Figure 8:**
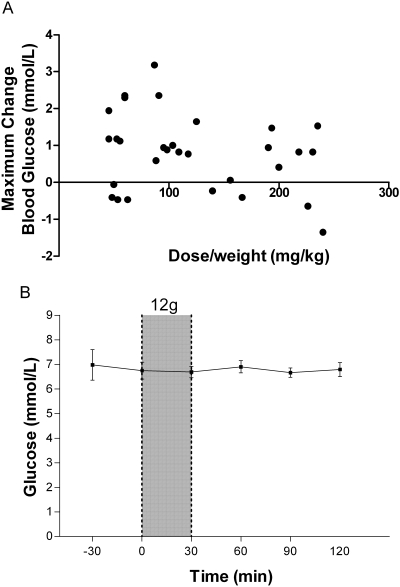
Effects of L-arginine hydrochloride infusion on blood glucose concentrations. Figure 8A: Relationship between dose/weight (mg/kg) of infused L-arginine and the maximal decrease in whole blood glucose concentration at any time during or in the two hours following infusion (n = 30; r = −0.27, p = 0.14). Figure 8B: Whole blood glucose concentrations of patients before and after 30 minute administration of the highest dose of L-arginine (12 g; n = 10). Shaded area indicates time of L-arginine infusion. Dots and bars indicate mean±SEM.

There was no change in lactate concentrations following infusions of any dose; and no dose-weight association (p = 0.7; **Figure 6A**). There was no significant change in the anion gap before and immediately after infusion at any dose, and no association was seen between the dose-weight of L-arginine given and the maximum change in anion gap (p = 0.9; **Figure 6B**). There was no correlation between dose (either mg/kg or dosage group) and the maximum change in phosphate (**Figure 7A**) or glucose **(** **Figure 8A** **)**. In addition, no significant changes were found in mean phosphate (**Figure 7B**) or mean glucose (**Figure 8B**) concentrations following the 12 g L-arginine infusion. One patient who received 3 g of L-arginine had a decrease in blood glucose from 6.7 mmol/L to 3.2 mmol/L but did not reach the range considered hypoglycemic (<3 mmol/L). This occurred 60 minutes after the completion of the L-arginine infusion and 7 hours after starting intravenous quinine. No significant changes in blood glucose were seen in patients who received the larger doses of 6 g and 12 g. There were no significant changes at any dose in creatinine, creatine kinase and liver enzyme concentrations within 24 hours (data not shown). Table 2 shows the maximum changes in the biochemical paramenters up to 2 hours after the start of infusion compared to pre-infusion values in each dosing group. The time course of the mean changes in venous blood potassium, bicarbonate, pH, chloride, phosphate and glucose concentrations before and up to 120 mins after administration of the maximum dose of 12 g are shown in **Figures 2B**, **3B**, **4B**, **5B**, **7B** **and** **8B** **.**

## Discussion

We have previously reported that in adults with moderately severe malaria, L-arginine infusion is able to improve NO bioavailability and endothelial function [5], suggesting the potential for a similar beneficial effect if used as adjunctive therapy in severe malaria. We now show that relatively rapid infusion of a single L-arginine infusion was safe at doses of up to 12 g infused at a rate of 24 g/hour in adults with moderately severe malaria.

Our findings are in agreement with other studies showing transient, modest, clinically insignificant decreases in blood pressure using doses equal or higher to those used in our study [14], [22]. The fall in blood pressure with L-arginine infusion is thought to be related to an increase in vascular NO synthesis [22]. In adult patients with sepsis, bolus arginine infusion at doses of 0.2g/kg causes a transient but significant decrease in mean arterial pressure [23]. In contrast, slower rates of infusion (∼1.1g/hr over 3 days) in adult sepsis cause no hemodynamic effects [24]. These findings indicate that both the dose and rate of infusion of L-arginine determine its hemodynamic effects. Despite minor effects on blood pressure, the doses used in our study were still able to significantly improve endothelial function [5].

Although not clinically significant, L-arginine infusion caused modest, transient and, dose-dependent increases in chloride and decreases in bicarbonate and pH. These changes were not associated with changes in respiratory or pulse rate, or an increase in lactate or the anion gap, and were likely due to the hydrogen and chloride ion components of the L-arginine hydrochloride infusion. Our results are consistent with an experimental study of canine sepsis which showed that infusion of L-arginine hydrochloride resulted in a dose-dependent decrease in bicarbonate and pH with an increase in chloride but not in lactate or anion gap [25]. As the only commercially available intravenous formulation of L-arginine registered for use in humans, L-arginine hydrochloride contains 4.75 mEq of hydrogen and chloride ions per gram. In this pharmacokinetic, safety and proof-of-concept study, L-arginine hydrochloride was given at a relatively rapid rate of infusion. If given as adjunctive therapy in severe malaria, L-arginine hydrochloride would likely be given less rapidly as a prolonged infusion which is more likely to allow compensatory mechanisms to ameliorate any effects of the hydrochloride on acid-base status.

Renal impairment is common in adult severe malaria. In considering the risk of acidosis in patients with renal failure, it is noteworthy that rapid infusion of higher doses of L-arginine hydrochloride (30 g over 30 mins) in patients with end stage renal failure resulted in no change in arterial pH [26]. In healthy subjects, L-arginine infusion improves renal blood flow and glomerular filtration rate [27], [28]. We found no evidence for an adverse effect of L-arginine on renal function in patients with moderately severe malaria, who by definition had normal renal function. Intravenous L-arginine has also been shown to have no deleterious effects on renal blood flow or glomerular filtration rate in patients with renal impairment [28].

As with other cationic amino acids, intracellular uptake of L-arginine results in displacement of intracellular potassium into the extracellular fluid [15]. Increases in plasma potassium following L-arginine infusion are well-described [29]. Mean increases in plasma potassium of 1.23±0.17 mmol/L (33%) have been described in eight normal adults following high dose (30 g; ∼0.5 g/kg) infusion over 30 minutes [16]. The risk of clinically significant hyperkalemia with this relatively rapid high dose infusion is higher in patients with underlying renal failure, particularly those with baseline hyperkalemia and/or concurrent spironolactone therapy [26]. The increase in potassium in patients with moderately severe malaria who received up to 12 g was transient and clinically insignificant. In a subgroup of African children with both severe malaria and acidosis, hyperkalemia (K^+^>5.5 mmol/L) was found in 16%, and was associated with increased mortality [30]. In adults, only 1 (2%) of a group of 51 consecutive patients with severe malaria defined by modified WHO criteria had a potassium level >5.5 mmol/L on admission [5]. However, future initial phase 2 studies in severe malaria will necessitate exclusion of hyperkalemic subjects, slow infusion rates and caution with patients with renal failure. Subclinical hypophosphatemia has been described in falciparum malaria [31], and clinically insignificant hypophosphatemia has been seen with rapid infusion higher doses (0.5 g/kg) of L-arginine [16]. However, there were no significant changes in plasma phosphate concentrations in the doses used in this study.

Because L-arginine increases the secretion of insulin [17] review articles have stated that L-arginine may cause hypoglycemia [14]. However there have been no published case reports or series to suggest this, likely because L-arginine also increases glucagon production [17]. To our knowledge, the only clinical trial reporting significantly altered blood glucose following L-arginine infusion described a significant *increase* in mean blood glucose following a dose of 0.5 g/kg [32]. In our study, one patient who received the lowest dose (3 g) experienced a decrease in blood glucose but not to the hypoglycemic range. Decreases in blood glucose were not seen in patients who received the larger doses of 6 g and 12 g. The patient was simultaneously receiving quinine infusion, a drug well known to increase insulin secretion and reduce blood glucose, even in non-severe malaria [33]. With the replacement of quinine by artesunate as treatment of choice for severe malaria, the risk of hypoglycemia in severe malaria has been significantly reduced [1]. The lack of a dose-response of L-arginine on glucose in our series, an alternative explanation for a fall in one patient, the use of artesunate in severe malaria, and the hyperglycemic effect of higher doses of L-arginine in other series [32], all suggest that adjunctive L-arginine is unlikely to have hypoglycemic effects in phase 2 studies in severe malaria.

Transient pain at the infusion site seen in 7% of patients in this study has also been described in other settings and is likely related to the hypertonicity of the L-arginine solution. Other rare adverse effects reported as single case reports in the literature were not seen in this study including extravasation reactions [34] and anaphylaxis [35].

There are limited data on the safety and efficacy of adjunctive L-arginine monotherapy in other severe infections in animals and humans [23]–[25], [36]. Studies of intravenous L-arginine infusion in patients with sepsis [23], [24] have reported hemodynamic but not biochemical or clinical endpoints. Trials of enteral preparations containing L-arginine in critically ill patients have shown conflicting results [37], [38]. The uncertain contribution of other components of enteral formulae limits the interpretation of the effects of L-arginine. With clear differences in pathophysiology [5], [39]–[41], safety and efficacy findings in other infections are difficult to extrapolate to severe malaria in humans although studies are ongoing to address this.

A parallel efficacy study performed in the same patients reported here [5], has shown that L-arginine increases NO production and restores endothelial function in falciparum malaria and may have the potential for a beneficial role as adjunctive therapy in severe malaria. The safety data in this study demonstrate that a single relatively rapid intravenous infusion of up to 12 g has no clinically significant hemodynamic, biochemical or local adverse effects in patients hospitalized with moderately severe malaria. Trials of L-arginine as adjunctive therapy can be extended to phase 2 studies in adults with severe malaria, where careful assessment of safety will also be required.

## Supporting Information

Protocol S1 Trial Protocol.
(0.53 MB DOC)
