# Supplementary material for: Safety Profile of L-Arginine Infusion in Moderately Severe Falciparum Malaria
Source: PLoS One. 2008 Jun 11;3(6):e2347. doi: 10.1371/journal.pone.0002347 (PMC2405947; doi:10.1371/journal.pone.0002347)
Supplement: Protocol S1 — Trial Protocol. (0.53 MB DOC) [file pone.0002347.s001.doc]

**Pharmacokinetics, pharmacodynamics, safety and preliminary efficacy of L-arginine in uncomplicated falciparum malaria (UM)**

**October 25 2004**

**Investigators:**

**Dra. Retno Gitawati** MS, Apt. (Research Pharmacist, Pharmaceutical and Traditional Drug Research and Development Center, NIHRD): Indonesian PI

**Prof Nick Anstey** MBBS PhD (Infectious Diseases Physician, Menzies School of Health Research [MSHR]) Australian PI

**Dr Tsin Yeo** MBBS (Infectious Diseases Physician and PhD student, MSHR)

**Dr Steve Duffull** PhD (Pharmacometrician, School of Pharmacy, University of Queensland)

**Dr Emiliana Tjitra** MD PhD (Senior Researcher, Communicable Diseases Research and Development Center, NIHRD)

**Dr Sanjaja** PhD (Senior Researcher, Food and Nutrition Research and Development Center, NIHRD)

**Dr Enny Kenangalem** MD (Research Clinician, NIHRD-MSHR Research Secretariat, Timika and Dinas Kesehatan, Timika),

**Dr Denny Takaendengan** MD (Research Clinician) and **Dr Paul Harijanto** MD (Head, Tropical Diseases, Universitas Sam Ratulangi, Manado)

**Dr Ric Price** MD (Senior Lecturer, Menzies School of Health Research)

**Dr Julie Simpson** PhD (WHO Statistical consultant)

**Consultants:**

**Prof David Celemajer** MBBS PhD (University of Sydney): non-invasive vascular function studies

**Prof Don Granger** MD**, Dr Bert Lopansri** MD (University of Utah Medical Center): HPLC

**Dr Graeme Maguire** MBBS PhD and **Prof Michael Pain** MD (Menzies School of Health Research and University of Melbourne): pulmonary physiology

**Prof Peter Sly** MBBS PhD (University of WA): exhaled NO

**Prof Roland Stocker** PhD (University of Sydney): oxidant measurements

**Safety Monitoring Committee (SMC):**

**Dr Peter Morris** MBBS PhD (Clinical Trials Researcher, MSHR): Chair

**Dra. Nani Sukasediati** MS, Apt. (Senior Pharmacist, Pharmaceutical and Traditional Drug Research and Development Center, NIHRD)

**Dr Paulus Sugiarto** MD (Clinical Director, RSMM, Timika)

**Dr Paul Kelly** MBBS PhD (Clinical researcher, MSHR)

**Dr Julie Simpson** PhD (WHO Clinical Trials Statistical consultant)

**Summary**

Between 12-30% of patients with severe malaria die despite rapid killing of malaria parasites with antimalarial drugs. This is because malaria parasites have already caused organ/tissue damage Better treatments are needed to reduce this unacceptably high mortality rate. One strategy to reduce mortality is to use adjunctive treatments (ie given in addition to standard antimalarial drugs) that will reduce/reverse the tissue damage caused by severe malaria. We have previously shown that levels of nitric oxide (NO) and its precursor arginine are low in severe malaria. NO production has been associated with protection from severe malaria in multiple human studies, including the NIHRD-MSHR studies in Indonesia. L-arginine is a naturally-occurring amino acid found in plant and animal protein. It is the only substrate for NO production. Levels of arginine are low in both children and adults with severe malaria, and very low levels have been associated with increased mortality. Because NO has multiple anti-disease properties (reducing cytokine-damage, reducing stickiness of blood vessels, reducing apoptosis), a treatment that will return NO levels back to normal in severe malaria, may improve outcome by reducing/reversing the tissue damage already caused by malaria parasites. We propose that by giving arginine in malaria, the low levels of arginine can be corrected and NO production can be returned to normal, allowing NO to reduce tissue and organ damage. Intravenous L-arginine has been used safely in large doses for over 40 years as a diagnostic test of pituitary function. It has also been used in patients with sepsis, coronary artery disease, peripheral vascular disease, sickle-cell vaso-occlusive crisis with improvement in NO production, vascular function and their clinical condition. These studies also show L-arginine infusion to be cheap and virtually free of adverse reactions, even at doses much higher than we will use in this study. **Our goal** is to determine if L-arginine has potential to reduce tissue damage and improve outcome in severe malaria when added to standard quinine therapy. As the first steps, we plan 3 stages over 3 years:

***Stage 1: Natural history study: time course of recovery of hypoargininemia (and pharmacodynamic measures) in uncomplicated and severe malaria***

**Aim:** Stage 1 is designed to determine the natural rate of recovery of L-arginine in patients with uncomplicated and severe malaria. Results will be combined with those from stage 2 to enable us to derive a pharmacokinetic (PK)-pharmacodynamic (PD) model using a computer software program which will accurately describe and predict the relationship between L-arginine plasma concentration and NO concentration both in plasma and urine as well as real time measurements of NO production in lungs and endothelium. This will enable us to determine the optimal dosage of L-arginine which should be given in future studies in malaria.

**Methodology:** We will undertake serial clinical and parasitological assessments on patients with uncomplicated or severe malaria, with serial measurements of blood (total ≤20ml) and urine (for arginine and NO measurements), along with measurement of exhaled NO (a suimple procedure breathing into a tube), endothelial function (non-invasively using an ultrasound method) and lung physiology (non-invasive breathing tests). Stage 1 is strictly observational with no administration of arginine and will be the equivalent to a “zero-dose” of arginine.

**Results:** We expect that there will be significant differences in both the initial levels and rates of recovery of arginine levels in the uncomplicated and complicated malaria and combined with Stage 2 will allow us to decide the optimal dosage and timing of L-arginine supplementation.

***Stage 2: Pilot pharmacokinetic, pharmacodynamic and safety study of arginine in uncomplicated falciparum malaria.***

**Aim:** This study together with stage 1 will allow us to determine the optimal dosage of L-arginine to improve production of NO, and confirm the safety profile of arginine in malaria.

**Methodology:** In a dose-escalation design, patients with *uncomplicated malaria* will receive dose of 3g of intravenous L-arginine, increasing to 6g then 12g (as well as standard quinine). As in stage 1 we will undertake serial clinical, parasitological over 24 hours with serial measures of blood (total ≤25ml) exhaled NO and ultrasound endothelial assessments for PK-PD measurements.

**Results:** We expect that arginine supplementation in malaria will increase NO production and be confirmed to be safe. Combined with stage 1, will allow us to decide the optimal dosage and timing of L-arginine supplementation.

***Stage 3: Randomised concentration controlled trial, safety and PK-PD study in uncomplicated malaria.***

**Aim**: To confirm that the loading and maintenance doses of L-arginine calculated from stage 1 and 2 will increase NO and improve biomarkers of outcome in uncomplicated malaria.

**Methodology**: Patients with *uncomplicated malaria* will be randomized (in addition to standard quinine treatment) to receive either IV L-arginine or saline, to determine if arginine administration results in more rapid return to normal of NO production and more rapid improvement in biomarkers of outcome (including lactate levels), endothelial function, fever clearance time and parasite clearance.

**Results**: We hypothesize that patients receiving L-arginine will have increased measures of NO production, improved biomarkers, and faster fever resolution time. We also expect further confirmation of the safety of L-arginine infusion.

***Expected Benefits:*** The results of the first 3 stages will confirm safety of arginine in malaria, and will establish an optimal dose that will increase NO production. We will test if arginine can improve a variety of biochemical and clinical markers of outcome. With the results of the current proposal, we will then be able to proceed to pharmacokinetic-pharmacodynamic studies in severe malaria with a view to a future randomized controlled trial in severe malaria. Arginine is a cheap, safe, natural product. Our longer term goal is to determine if adding arginine to standard antimalarial therapy in severe malaria, can reduce tissue damage and further decrease mortality in severe malaria.

**Background**

Please see appendix 1 for the list of abbreviations

**1. Wellcome Trust-NHMRC studies:**

In 2003, NIHRD and MSHR submitted a joint funding submission to The Wellcome Trust (a UK-based medical charity) and NHMRC in response to their call for submissions for their inaugural International Collaborative Research Grants Scheme. The submission was endorsed by the Secretary of NIHRD and the Director of MSHR. Over 140 submissions were received, and these were scrutinized by an international scientific panel for both scientific merit and potential for translation into improvements in health. The NIHRD-MSHR submission was one of only 11 that were successful. There were two components to the submission: The first involved comparative studies of artemisinin combination therapy in Timika, and whether widespread deployment will reduce malaria morbidity and mortality in a medium-transmission region. The ACT comparative studies have been approved by the Ethics committee in Jakarta and the studies have commenced in Timika. The second component of the Wellcome-NHMRC submission is an extension of the NIHRD-MSHR nitric oxide studies. For the first time we will extend the findings from our pathophysiology studies to test whether a simple intervention with arginine, a natural product found in food protein, can increase nitric oxide production and reduce malaria morbidity when used as adjunctive treatment for malaria.

**Post-graduate training opportunities:**

The Wellcome-NHMRC studies provide postgraduate training opportunities for each of the core partners involved: NIHRD, MSHR and Sam Ratulangi University in Manado.

These postgraduate training activities are outlined in the original submission endorsed by the Secretary of NIHRD in 2003, and are shared among the submitting partners: ie.

1) NIHRD: has/will have 2 postgraduate students [both ACT studies]

2) MSHR has/will have 2 postgraduate students [one ACT, one arginine study].

3) Universitas Sam Ratulangi (Manado), has had 2 clinical postgraduates working on the previously-approved SEAQUAMAT study as the clinical research requirement for their post-graduate qualification in internal medicine. From 2005, internal medicine trainees will undertake components of the arginine study for their internal medicine research projects.

**Background to NO-arginine studies:**

*How can we reduce mortality in severe malaria?*

Mortality in severe malaria remains between 12-30% despite the best available antimalarials^1,2^. There are two goals in designing better treatments to reduce mortality in severe malaria: 1) to kill parasites faster and 2) to decrease the excessive inflammation/tissue damage found in severe malaria.

To address the first goal, we are currently conducting a study at RSMM, Timika looking at whether drugs that kill parasites more quickly will reduce mortality [the multi-centre SouthEast Asian trial of artesunate vs quinine (SEAQUAMAT)].

To address the second goal, this arginine-malaria protocol describes the first stages of studies that seek to determine if arginine can act to dampen the excessive inflammation found in severe malaria.

*Arginine and NO:* Arginine: is a naturally occurring amino acid found in plant and animal protein (eg nuts). It is the critical substrate required for the generation of nitric oxide in the body by the action of a family of enzymes the nitric oxide synthases (NOS)^3^. The constitutively-expressed enzymes NOS1 and NOS3 produce low-level homeostatic neuronal and endothelial NO, whereas in inflammatory states inducible NOS (NOS2) generates high level NO production in a variety of cell types^3^, including peripheral blood mononuclear cells (PBMCs)^4^. NO has antimicrobial and immunomodulatory actions during inflammation^4-6^. Generation of NO from intracellular NOS is critically dependent upon transport of extracellular arginine into cells via the cationic amino acid transporters, CAT1 and CAT2, with the K_m_ for uptake of arginine being 70-150 uM^7^. In situations of inadequate extracellular arginine availability, NOS generates superoxide instead of NO^8-10^.

*Arginine: clinical pharmacology:* In healthy humans endogenous arginine is a non-essential amino acid, synthesised from citrulline, which is in turn synthesised from enteral glutamine and glutamate^7^. However, in acute inflammation such as in sickle cell crisis^11,12^, severe burns^13^ and sepsis^14^, plasma levels of arginine are low and arginine is “conditionally essential” ^13,15^. Arginine is metabolised by arginases (in hepatocytes and other cell types) to form urea and ornithine, but there is strict segregation of hepatic and plasma arginine pools and only 5% of urea production is derived from plasma arginine^7^. Arginine is also renally excreted but undergoes almost complete tubular reabsorption^16^. When exogenous arginine exceeds the renal threshold for tubular reabsorption (eg in supraphysiological dosage), there is additional renal excretion^16^. The pharmacokinetics of L-arginine are derived from studies of arginine given in large doses only to adults with normal plasma arginine concentrations, where it has been described by non-compartmental analysis^16,17^. There is apparent evidence for non-linear pharmacokinetics, where a dose-dependent elimination half-life has been reported^16,17^. The reported half-life for oral and IV arginine ranges from 40-120 minutes (with variability due in part to the non-linearity).^16^ Reported clearance (CL) ranges from 544-1018 ml/min^16^. In subjects with normal plasma arginine concentrations, the relationship between pharmacokinetic and pharmacodynamic data (urine NOx and total peripheral resistance) was best described using a linear direct effect model, in which effect was linked directly to L-arginine plasma concentration^17^.

*Nitric oxide and severe malaria:* Our studies over the last decade have associated NO production with protection from severe malaria. In African children we found an inverse association between malaria disease severity and systemic NO production/monocyte NOS2 expression^18^. In longitudinal studies, blood monocytes from children with past severe malaria had less NOS activity than those from children with past uncomplicated malaria^19^. We have also found novel NOS2 polymorphisms associated with increased NO production and protection from severe malaria^20^. In Papua, we have now recruited over 600 adults with and without severe malaria and shown the same inverse association between disease-severity and NO/PBMC NOS2 in *non-immune* *adults* from *different ethnic groups* in a region of *very different malaria epidemiology* (^21^and manuscript in preparation; see fig 1). Potential mechanisms for these associations include the ability of NO to decrease pro-inflammatory cytokines^22^, endothelial cell adhesion molecule expression^23^, and parasite cytoadherence (our unpublished data) ^20^. In complementary immunohistochemical studies, other investigators have shown that in contrast to suppression of PBMC NOS expression/systemic NO production, tissue NOS expression is increased in severe malaria^24^. These tissue studies are unable to directly quantitate NO production. We hypothesise that inadequate arginine levels found in severe malaria limits tissue and monocyte NO production; and that by generating superoxide NOS exacerbates deleterious oxidant stress in severe malaria.

**Figure 1. NO and NOS parameters measured in Papuan adults.** Urinary NOx, PBMC NOS enzyme activity, PBMC NOS2 antigen by immunoblot, and NOS2 mRNA (by RT-PCR) were all inversely associated with disease severity. RHC: rural healthy controls, UHC: urban healthy controls, UM: uncomplicated falciparum malaria, SM: severe malaria

*Hypoargininemia in severe malaria: does this limit NO production and increase mortality?* In Tanzanian children we have now shown that arginine concentrations are inversely associated with disease severity (fig. 2). Moreover, in both univariate (OR 4.0 [95% CI 1.1-15.2]) and multivariate analysis (OR 26.2 [95% CI 1.2-586])], L-arginine level below the median in Tanzanian children with CM was associated with mortality^25^. In Indonesian adults, the same inverse association between arginine and disease severity is found (fig. 2)


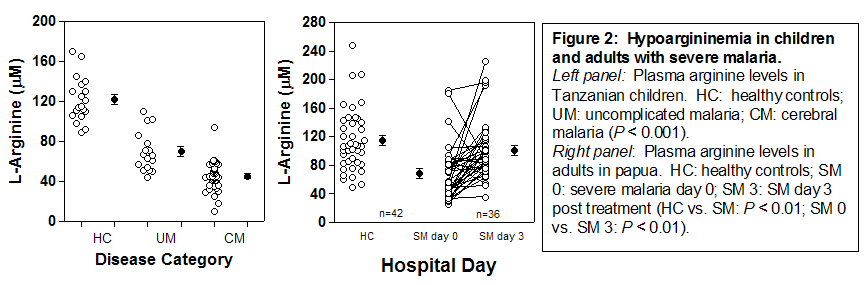


Plasma arginine in healthy subjects was not significantly different among different age groups or different ethnic groups^25^. Our data have shown a mean plasma arginine level for healthy Tanzanian children (n=19) of 122 uM (lower limit of normal 78uM). Mean plasma arginine in American adults (n=23) is 117 uM with lower limit of normal of 59uM^25^. In Indonesian adults in our previous studies in Jayapura (n=43), mean plasma arginine was 114 uM with lower limit of normal of 54 uM (Anstey, Tjitra et al, unpublished data). Hypoargininemia can therefore be defined as levels below 54uM (the lower limit of normal in healthy Jayapura residents). In the Tanzanian data above, there were no differences in fasting or weight-for-age among groups and levels returned to normal by day 3 in adults with severe malaria, despite continuing anorexia. This suggests that our arginine findings are not related to underlying malnutrition or to inadequate dietary arginine ingestion prior to malaria^25^. The causes of low NO production/monocyte NOS2 and low arginine in severe malaria are likely to be multi-factorial^25^. In separate studies, we are examining cellular mechanisms of hypoargininemia, including arginase activities and PBMC levels of enzymes involved in synthesis, transport and degradation of arginine. Cytokine responses decreasing NOS2 also diminish factors involved in cellular uptake and biosynthesis of L-arginine.^7^ Non-plasmodial parasites activate arginases during infection^26^ but whether *P. falciparum* activates arginases is not yet known. Metabolic turnover may be increased in malaria, similar to that seen in burns^13^ and sepsis^15^. ***Irrespective of its cause we hypothesise that hypoargininemia limits NO production in malaria and may contribute to severe disease.*** With intracellular NOS activity being critically dependent on adequate circulating L-arginine, and with arginine levels in SM well below the K_m_ for cellular uptake of arginine^7^ hypoargininemia is likely to result in sub-optimal monocyte, endothelial, and tissue NO production in SM. Moreover, hypoargininemia may enhance^8-10^ the deleterious oxidative stress described in SM^27,28^. Strategies to increase NO production (such as L-arginine administration) may improve outcome by ameliorating endothelial dysfunction and decreasing deleterious inflammatory cytokine and oxidative responses. Moreover L-arginine reduces ischaemia-reperfusion injury^29^, and kills *P. falciparum* *in vitro*^30^ and *in vivo*^31^ through an NO-mediated effect. Although hypoargininemia can be defined as levels below 54uM (the lower limit of normal in healthy Jayapura residents), increasing plasma levels within and above the normal range will be expected to increase NO production. However, levels below 600uM (4X Km for CAT transporters) will result in submaximal arginine uptake into cells, and 600uM will therefore be the plasma level for therapeutic target.

*Clinical use of arginine in humans:* L-arginine has been safely used in humans for decades^16,32^. Arginine has also been well tolerated when given short term (6-30g IV/orally) and long term (6-12 g/day orally) in healthy subjects, and subjects with severe cardiovascular disease and cystic fibrosis with minimal toxicity^16,17,32-35^. In these diseases, arginine increases NO metabolites and exhaled NO, with improvements in endothelial function, perfusion, symptom scores and exercise tolerance^16,17,33,34^. Use of 12g IV arginine in sepsis has been shown to improve cardiac output and improve oxygen transport^35a^. L-arginine therapy corrects the hypoargininemia and impaired NO production found in patients with sickle cell vaso-occlusive syndrome (SCD VOCS),^12^ a syndrome with endothelial dysfunction and impaired NO production similar to malaria^11^. Pharmacokinetic data have not been reported in SCD^12,36^. A randomised trial of L-arginine in SCD VOCS is in progress with no significant adverse effects using 0.1 g/kg IV 8 hrly (C. Morris, personal communication, 2004). Use of enteral arginine supplementation (with or without other “immunonutritional” supplements) in critically unwell intensive care unit patients has been associated with reduced inotrope requirements, higher base excess, reduced risk of nosocomial infection, reduced ventilator days and reduced hospital stay,^37-40^,^41^. Beneifts were particularly noteworthy in septic ICU patients^41^. Use of oral arginine in patients with tuberculosis showed an increase in weight gain, reduced sputum conversion time and reduced symptoms compared to controls^41a^. Arginine is off-patent and available as an inexpensive, generic, GMP-standard product for human use. For potential adverse effects of arginine (in a malaria context) see “Study 2” below.

**3. Rationale for the Arginine-NO studies:**

There is a need for novel adjunctive antidisease therapies to reduce the unacceptably high case-fatality of severe malaria (SM) further^1,2^. Irrespective of its cause, hypoargininemia likely limits NO production and is associated with death in cerebral malaria^25^. Our NO-arginine-malaria studies and the results of arginine trials in related diseases suggest that arginine may have potential for therapeutic use in malaria. L-arginine is commercially available as an off-patent product for human use, is inexpensive, and has been widely used worldwide with little toxicity despite supra-physiological intravenous dosing as a diagnostic pituitary function test and administration in other human diseases^36^ (see also “clinical use of arginine in humans” above). It is important to determine whether arginine administered to malaria patients increases NO production, reduces measures of oxidant stress and has physiological effects at the doses proposed: this has implications for understanding the pathophysiology of severe malaria, as well as therapeutic implications. Arginine pharmacokinetics have not yet been described in hypoargininemic states such as uncomplicated and severe malaria. Our proposed safety, pharmacokinetic-pharmacodynamic and efficacy studies in uncomplicated malaria will be the first steps in cautiously determining if exogenous arginine administration has therapeutic potential in malaria.

**4. Our aims** are to:

4.1 Determine the natural history of recovery of plasma arginine levels and our pharmacodynamic measurements in uncomplicated and severe malaria.

4.2 Determine the pharmacokinetics, pharmacodynamics and safety of L-arginine in uncomplicated malaria**.**

4.3 Determine preliminary effects on biomarkers of outcome in uncomplicated malaria.

4.4 Establish the relationship between malaria disease severity and those measures used to monitor pharmacodynamic effects: exhaled NO, plasma/urine NO metabolites, oxidant stress, brachial artery dilatation, doppler evaluation of microvascular blood flow, physiological deadspace.

**We will test the following hypotheses arising from our work:**

Low plasma arginine levels in uncomplicated malaria limits nitric oxide (NO) production in malaria. Arginine supplementation is safe, restores plasma arginine levels to normal, increases systemic and exhaled NO production, reduces oxidant stress, and improves a number of physiological measures of relevance to malaria (including vascular endothelial function and physiological deadspace)

**5. Study Design:**

**Study site:**

Patients will be enrolled at RS Mitra Masyarakat (RSMM), Timika, Papua, Indonesia. A malaria research collaboration is already established with this hospital as part of the MOU between NIHRD, RSMM, Regional Ministry of Health and MSHR. It is the site of our recent NO-malaria studies, lung-malaria and SEAQUAMAT severe malaria studies all of which have involved capacity building and clinical research training to local investigators.

RSMM has approximately 200 malaria admissions per month The hospital has a well-equipped clinical research ward attached to its high dependency unit, where the arginine administration studies will take place.

**Overview:** We will determine pharmacokinetics, pharmacodynamics, safety, appropriate dosing and preliminary efficacy of L-arginine in uncomplicated malaria: the arginine studies will be comprise three stages: studies 1 and 2 will be undertaken in parallel, followed by stage 3.

1. *Natural history study: time course of recovery of hypoargininemia (and pharmaco-dynamic measures) in uncomplicated and severe malaria:*
2. *Pilot safety, pharmacokinetic and pharmacodynamic study of exogenous arginine in uncomplicated falciparum malaria*
3. *Randomised concentration controlled trial, safety and pharmacokinetic study in uncomplicated malaria*

**Goal:** to be able to optimize the arginine dose to achieve the best outcomes possible in malaria.

**Patients:**

*Study 1:*

*Uncomplicated falciparum malaria (UF)*:

Inclusion criteria:

1. age 18-60 years
2. *P. falciparum* parasitemia (1,000-100,000 parasites/ul).
3. Clinical syndrome consistent will malaria associated with documented fever (axillary temperature ≥ 38^o^C) or self-reported history of fever in the last 48 hours with no other cause present
4. Time of commencement of quinine known
5. An indication for hospital admission [eg relative cannot stay at home to look after them or supervise their treatment at home, one episode of vomiting (but not repeated episodes of vomiting)], but not having the warning signs or severe malaria criteria in “exclusion criteria” below
6. Informed consent obtained

Exclusion criteria:

1. pregnancy or lactation
2. mixed infection with *P. falciparum* and *P. vivax*
3. warning signs of altered mental state and inability to sit unaided
4. features of severe/complicated malaria^2^ (see “severe malaria” below)
5. diabetes
6. systolic BP <100 mmHg
7. serious underlying diseases (cardiac, hepatic, kidney and lung)
8. malnutrion
9. other infection
10. concurrent therapy/medication including: anti-TB, Asthma drugs.

All UF subjects will be treated with quinine as per standard RSMM practice. Height and weight will be documented in all subjects.

*Severe malaria (SM)*, defined by modified WHO criteria^42^:

Inclusion criteria:

1. age ≥ 18 years
2. Informed consent obtained
3. Time of commencement of quinine known
4. any level of *P. falciparum* parasitemia, and one or more of the following criteria:
   1. acute renal failure (creatinine >265umol/L)
   2. hyperbilirubinemia (total bilirubin > 50 umol/L) with either renal impairment (creatinine > 130umol/L) or parasitemia of >100,000 parasites/uL
   3. blackwater fever
   4. hyperparasitemia (>10% parasitised red cells)
   5. cerebral malaria (Glasgow coma score <11)
   6. Hypoglycemia
   7. Respiratory distress (RR>32)
   8. Acidosis: venous bicarbonate < 15 mmol/L.
   9. Shock: systolic BP <80 mmHg with cold peripheries

Exclusion criteria:

1. severe anemia (Hb <6 mg/dL) and
2. pregnancy or lactation.

Clinical care, and monitoring will be as per standard national, RSMM and WHO protocols^2^.

*Studies 2 and 3: Uncomplicated falciparum malaria (UF)*:

Inclusion criteria:

1. age 18-60 years
2. *P. falciparum* parasitemia (1,000-100,000 parasites/ul).
3. Clinical syndrome consistent will malaria associated with documented fever (axillary temperature ≥ 38^o^C) or self-reported history of fever in the last 48 hours with no other cause present
4. Commenced oral quinine ≤18 hours prior to scheduled commencement of arginine
5. An indication for hospital admission (eg relative cannot look after/supervise treatment at home **but not having any warning signs or severe malaria criteria in “exclusion criteria” below)**
6. Informed consent obtained

Exclusion criteria:

1. pregnancy or lactation
2. mixed infection with *P. falciparum* and *P. vivax*
3. Warning signs of altered mental state and inability to sit unaided
4. features of severe/complicated malaria^2^ (see “severe malaria” Study 1 above),
5. diabetes
6. systolic BP <100 mmHg
7. serious underlying disease (cardiac, hepatic, kidney)
8. initial iSTAT test showing any of the following values:
   - 1. glucose < 4 mmol/L
     2. K+ ≥ 4.2 meq/L
     3. Cl- > 106 meq/L
     4. HCO3- < 20 meq/L
9. known allergy to L-arginine
10. concurrent therapy with any of the following medications:
    - 1. spironolactone,
      2. oral nitrates,
      3. phosphodiesterase inhibitor (eg sildenafil [Viagra])
      4. alpha-blocking antihypertensive agents (eg prazosin)
      5. L-arginine

All UF subjects will be treated with quinine as per standard RSMM/Depkes protocols and will commence arginine within 18 hours of commencing quinine. Height and weight will be documented in all subjects.

**Study 1**

***Time course of recovery of plasma arginine, NOS cofactors and pharmacodynamic measures in uncomplicated and severe malaria:*** Our data on existing cryopreserved plasma in Indonesian adults indicate that plasma arginine levels in severe malaria have returned to normal by day 3 (fig. 2), however the rate of recovery of arginine levels in uncomplicated and severe malaria is not known. In 20 patients with UF and 20 with SM (defined above), we will measure serial measurements of plasma arginine to determine the natural history of recovery of plasma arginine concentrations in patients with malaria, and the natural history of recovery of the pharmacodynamic measurements to be used in stage 2.

*Clinical measures.* Vital signs (pulse rate, BP, temperature, respiration rate), and a standardised clinical assessment (symptom/signs listed in Table footnotes) will be serially monitored over 24-72 hours (see table 1). ECG will also be monitored from 0-240 mins.

*Sampling regimen and amino acid measurements:*

1. Blood: An additional venous cannula for sampling will be placed at the time of enrolment. At the time of cannula insertion we will take 10ml heparinised baseline blood (processed as per enrolment in NO Study II SOP for heparinised blood).

Subsequent sampling regimens:

2 ml (in heparinised blood) collected on up to five further occasions over 48-72 hours depending on clinical need for hospitalisation (see example of sampling schedule in table 1). **Of particular note, for the pharmacokinetic-pharmacodynamic modeling proposed in studies 1 and 2, it is advantageous for serial blood samples/measures to be collected at varied time points.**

Total amount of blood taken: ≤20 ml.

Plasma will be cryopreserved at -70ºC for later quantitation of the following:

- arginine, its metabolites and related amino acids by HPLC^25^ (Shimadzu) using our established methodology (figure 2).
- NO metabolites (nitrate plus nitrite) using Greiss reagents as we have performed previously, and measures of oxidant production (including oxidized glutathione).
- CK, LFTs, lactate, urea, creatinine and the electrolytes required for study 2 below.
- sICAM-1 and other biochemical measures of endothelial activation

See footnotes to table 1 for where each test will be measured.

**Please see Appendix 2 for details of the type of specimens, type of tests and location of where the tests will be performed.**

2. Urine: will be collected on admission and whenever this is passed whilst in hospital for serial measures of NO and pterin metabolites. All urine collections, stabilization and processing will be done in dim light. Samples are not filtered or centrifuged because pterin species may be associated with particulate material in the urine^43^. Freshly obtained urine shielded from light will be separated into two samples: one for NO metabolites; the other for pterins which will immediately stabilized by acidification to pH 1 with one drop of 6 N HCl and then immediately frozen. Urines are later thawed at 40^o^ C and processed for pterins. The oxidized samples are then frozen at -150 ^o^ C for storage until analysis by HPLC at MSHR, Darwin.

3. Exhaled NO*:* We will also measure serial exhaled NO online at up to 6 timepoints over 24-72 hours (see example in table 1), as per exhaled NO SOP (Appendix 4) using ATS Guidelines^44^ We will be using on-line measurements using the NIOX NO analyzer. The advantage of this apparatus is that all that is required is a simple blow into a mouthpiece, without complex instructions and without complex breath holding instructions etc.. This analyzer has recently been licenced by the US FDA for clinical use in asthmatics, including children as young as 4 years old.

**See Appendix 4 for the standard operating procedures for measurement of exhaled NO.**

4. Physiological measures: In both UF and SM we will also serially quantify those physiological measures that will be used to assess pharmacodynamic effects of arginine in studies 2 and 3: brachial artery dilatation, doppler evaluation of microvascular blood flow, gas transfer (DLCO), ECG and physiological deadspace .

*4.1 Non-invasive vascular studies:* As additional pharmacodynamic measures, we will also use non-invasive vascular sonography to assess the impairment during malaria of endothelial function by brachial artery dilatation^45-48^, peak reactive blood flow by Doppler assessment and finger plethysmography.

Nitric oxide production by endothelial cells can be assessed by monitoring brachial artery reactivity to ischemia-promoting stimuli such as an inflated blood pressure cuff to assess endothelial production of nitric oxide. A baseline study of brachial artery ultrasound will be performed with the arm in the supine position. Arterial diameter and flow velocity will be determined. A cuff will be placed over the forearm (between the elbow and hand), distal to the ultrasound probe. Measurements will be performed at rest. The cuff will be inflated to 250 mmHg for 4.5 minutes and then released. Further ultrasound measurements will be performed after cuff deflation, and after 15 minutes of rest. In addition, peak reactive blood flow (a measure of distal microvascular blood flow) will be estimated by Doppler analysis immediately after cuff release. Finger plethysmography can measure both pulse rate as well as endothelial function. It causes no discomfort and requires just the placement of a sensor on the finger (similar to a finger pulse oximeter).

These will be measured up to five times as per example in table 1, to assess serial changes (and a hypothesized improvement) in endothelial function following treatment of malaria.

These measures are not painful. A cuff *above* the elbow can be unpleasant, but the technique we will use involves cuff inflation *below* the elbow, between the elbow and hand: this does not cause pain. The subject may experience very minor discomfort during inflation of pressure cuffs during the studies. This discomfort will be transient and would not be worse than having blood pressure measurement in routine clinical practice.

*Rationale for brachial artery studies:* the brachial artery is not the target in severe malaria, but the endothelial cell is. The brachial artery studies allow us to non-invasively measure of two key *endothelial* functions that are impaired in severe malaria:

*1. Endothelial dysfunction:* 80% of the brachial artery dilatation following cuff-release is due to nitric oxide production by endothelial cells in real time. In severe malaria, the endothelium of blood vessels is severely affected: endothelial function is impaired, the endothelium is activated and ICAM-1 and other endothelial receptors are significantly increased^49^. Parasitised red cells cytoadhere to endothelium in capillaries and post-capillary venules. We cannot measure endothelial NO function directly in capillaries and venules, but we can measure endothelial function in the brachial artery. There is no reason to think that endothelial cell impairment due to low arginine will be any different in the artery to the impairment in endothelial function from low arginine in the capillary or venule endothelium. Our hypothesis is that arginine will allow endothelial cells (arterial, capillary, and venules) to recover their ability to make NO. In has been shown *in vitro* that nitric oxide can reduce the amount of ICAM-1 expression and decrease adhesion of infected red blood cells to endothelial cells^50^. We can measure endothelial NO production directly with brachial artery dilatation technique. Measuring brachial artery dilatation will supplement our other indirect measures of nitric oxide and arginine in plasma and urine (total body NO), by giving us a real-time bedside measure of *endothelial* NO production^45-48^. There will be training in this technique to all investigators working on site in Timika.

*2. Microvascular blood flow:* the non-invasive Doppler ultrasound assessment of peak reactive blood flow in the brachial artery wave pattern *immediately after* cuff release is inversely related to distal microvascular blood flow. ie microvascular blood flow in the small vessels of the hand (which will be affected by parasitized red cell obstruction) can be measured by Doppler pattern upstream at the brachial artery immediately after cuff release. So, as well as the information on endothelial blood flow above, the brachial artery studies can give us important information about the degree of microvascular obstruction distally in the hand before and after arginine infusion. Microvascular flow is directly related to the degree of obstruction in severe malaria.

*4.2 Gas Transfer* (DLCO) and *physiological deadspace*: to examine effects on organ-specific function we will use our existing pulmonary physiology apparatus and procedures at RSMM (as per current HREC-approved lung-malaria studies [HREC 02/13]) to measure gas transfer and pulmonary deadspace up to 5 times as per example in table 1.

*Interpretation:* Data will be incorporated into pharmacokinetic-pharmacodynamic models [see study 2 below] as “zero dosage” of arginine. The baseline recovery of arginine levels in patients with malaria not receiving arginine is very important in the development of the PK-PD models. Serial measurements will also enable comparison of recovery times of arginine (and all the pharmacodynamic parameters) to be made between UF and SM controlling for the potential confounding effects of admission parasitemia, age, sex, and duration of fasting.

In order to have sufficient power to compare admission values of exhaled NO, vascular studies and physiological deadspace among disease categories, we will also measure these parameters on admission only in an additional 30 UM, 30 SM subjects (giving a total of 50 admission values in each group). This total sample size is based on the numbers required in our earlier NO-malaria studies to show a significant difference in NOS activity and arginine among the same groups of patients. We hypothesise that in severe malaria the physiological values and exhaled NO will be more impaired than in UM. In order to compare these values between uncomplicated falciparum malaria (in which cytoadherence to vascular endothelium occurs) and uncomplicated vivax malaria (in which cytoadherence to vascular endothelium does not occur) we will also undertake admission measurements of vascular function (as above), exhaled NO and physiological deadspace in 50 patients with uncomplicated vivax (UV) malaria (defined as per UF above, except for any level of *P. vivax* parasitemia on thick film in the absence of mixed infection). We hypothesise that these parameters will be more impaired in UF than UV.

All subjects will be treated with standard antimalarial drugs (currently quinine) as per standard RSMM/Depkes practice/protocols. Height and weight will be documented in all subjects.

**Please see Appendix 3 for the types of physiological tests that will be done and where they will be done.**

**Study 2**

***Pilot pharmacokinetic, pharmacodynamic and safety study of exogenous arginine in uncomplicated falciparum malaria:***

*Overview:* In this pilot study in uncomplicated malaria we will generate initial safety, pharmacokinetic and pharmacodynamic data for exogenous arginine administration. We will use a dose-escalation design; all doses (3-12g) will be in the low-mid range of dosages previously used safely in humans (6-30g). *Dosing:* All doses of arginine hydrochloride will be infused intravenously over 30 mins (which is the standard infusion time). Initial dosing will be 3g (n=10), increasing to 6g (n=10), increasing to 12g (n=10) if lack of significant toxicity is demonstrated at each dosing level. Each arginine dose will be added to 100ml normal saline (the standard diluent safely used in other studies) such that total concentration is ≤10% (see CRF)

In addition to arginine, all subjects will be treated with standard antimalarial drugs (currently quinine) as per standard RSMM/Depkes practice/protocols.

*Clinical measures.* Vital signs (pulse rate, BP, temperature, respiration rate), and a standardised clinical assessment (symptom/sign list) will be serially monitored over 48 hours (see table 2). ECG will be monitored from -60 to 240 mins.

*Blood sampling*:

Two venous cannulae will be placed:

A cannula in the right forearm will be used for administration of IV arginine, and for any IV fluids required after arginine administration.

A cannula in the left forearm or antecubital fossa for sampling of venous blood, placed one hour prior to arginine adminstration.

At the time of cannula insertion we will take 7 ml heparinised baseline blood. This will be the -60 minute sample: 60ul will be used for baseline iSTAT EC8+ measurement of electrolytes, glucose, urea, and bicarbonate. The remainder will be processed as per NO Study II SOP for heparinised blood.

Subsequent sampling regimen (from cannula): up to 18 ml (in heparin) over the next 20 hours, taken in up to 9 divided aliquots during this time interval. *Including the enrolment sample, this amounts to a maximum of 25ml taken over 21 hours.* For study 2, we will use a sampling protocol as outlined in table 2, with frequent initial samplings in an approximate geometric time series to include at least four half lives of arginine, plus one blood sample the following morning. These samples include serial measurements required for clinical management of malaria (including serial parasitemia, blood glucose), pharmacokinetics of arginine (arginine, its metabolites, and related amino acids), pharmacodynamic measures (NO metabolites and oxidant production, electrolytes, pH) and biomarkers of response to antimalarial treatment (including lactate, glucose and parasitemia [see example table 2]); and safety, including serial glucose, K^+^, Cl^-^, HCO_3_^-^, pH, PO_4_^3-^, and a final measurement at 20 hours for urea, creatinine, lactate, liver enzymes and creatine kinase.

We will also measure exhaled NO online at each of these time points, using ATS Guidelines^44^.

Please see attached ATS guidelines behind the protocol.

*Additional Physiological assessments:*

*Vascular studies:* As additional pharmacodynamic measures, we will also use serial non-invasive vascular sonography to assess effects on endothelial function by brachial dilatation, microvascular blood flow by Doppler assessment of peak reactive blood flow and finger plethysmography (see Study 1).

*Gas Transfer* (DLCO) and *physiological deadspace*: to examine effects on organ-specific function we will use our existing pulmonary physiology apparatus and procedures at RSMM (HREC 02/13) to measure gas transfer and pulmonary deadspace at intervals outlined in table 2.

*Interpretation:* Pharmacokinetic models will be fitted to concentration-time and concentration-effect data from studies (a) and (c) using NONMEM (v. 5)^51^, to provide population parameter estimates, mean parameter values and their between-subject variability where possible. This analysis will include assessment of possible covariate relationships, where parameter values such as clearance may be correlated with patient demographics such as age. A model will be developed that describes the time course of effects of arginine in terms of NO production (plasma NOx/creatinine ratio [systemic NO production] and exhaled NO [pulmonary NO production]), oxidant stress, endothelial function, microvascular flow and gas transfer/physiological deadspace. It is also intended to address biomarkers of effectiveness. **Dra Retno from NIHRD will be invited to the Dept Pharmacy in Brisbane to join the NONMEM modeling for the PK-PD relationships outlined above.**

Safety data from study 2 will be reviewed by an experienced independent data monitoring committee (see appendix) before proceeding to study 3. The pharmacokinetic-pharmacodynamic model developed in study 2 will enable us to predict the dosing and best sampling schedule (both pharmacokinetic and pharmacodynamic) for use in study 3. If the pharmacokinetic-pharmacodynamic model suggests that a higher dose than 12g may be required, a dose of up to 18 g may tested in stage 2 of the study prior to commencing stage 3.

*Potential adverse effects (in study 2 and study 3) and study response:* A number of adverse effects have been previously noted with large *supraphysiological* dosing in subjects with normal plasma arginine levels. These have been infrequent and usually at the standard high dose (≥30g) required to stimulate growth hormone release, **a *dose that will not be used in our studies.*** Arginine is a natural product that has been widely used in subjects with cardiovascular disease, sickle cell disease, and sepsis, with an excellent safety profile. This study primarily examines pharmacokinetic-pharmacodynamic modeling, but will also confirm safety in uncomplicated malaria.

**Patients with uncomplicated malaria in Indonesia have mean levels of arginine *below* normal, and combined with a dose well below the standard 30g IV dose used in other settings, we do not expect significant adverse effects to occur. Whilst we believe the following adverse effects to be very unlikely, our protocol allows us to detect, and if necessary rectify them:**

**Possible Adverse Effects and Monitoring and Rescue Treatments Protocols:**

**1. Adverse Effects Documented With Evidence**

*a) Effects on blood pressure:* Most patients receiving arginine have no significant effect on blood pressure. Any effects on blood pressure are usually minimal, even with the 30g IV dose used clinically, much higher than we expect to use in this study^16,17^. Endothelial NOS expression in severe malaria^24^ makes it important we monitor this closely. Arginine supplementation in sepsis has not significantly exacerbated hypotension^41^ despite demonstrated tissue NOS expression in sepsis^52^. In previous studies, any effect on blood pressure has been minor and seen during the infusion, returning rapidly (within minutes) to normal as soon as the infusion is finished. As outlined in table 2, to monitor for hypotension, the subject’s vital signs including blood pressure will be taken every 10-15 minutes prior to infusion, every 5-10 minutes during the infusion and for 15 minutes after the end of the infusion, then 10-15 minutely for the rest of the first hour after the end of the infusion, and half hourly for the next two hours, then 2-4 hourly thereafter.

Response: If systolic blood pressure falls >25 mmHg or below 80mmHg or if the attending clinician is clinically concerned, the arginine infusion will be stopped and legs elevated. Previous studies using higher doses of arginine than we will use in this study have shown the BP rapidly returns to normal within minutes of stopping the arginine infusion, and this is expected to be all that is required. If SBP persists < 80 mmHg, 0.9% saline will be infused at a rate based on the treating clinician’s clinical judgement.

*b) Electrolyte abnormalities:*

- *Effect on K+:* Hyperkalemia may result from redistribution of K^+^ from intracellular fluid to extracellular fluid. In seven of eight normal Italian subjects receiving high dose arginine (30g IV), mean plasma K^+^ transiently increased by 1.23± 0.17 meq/L (range 0.6-1.7), peaking at 60-90 mins after start of a 30 min IV infusion^54^. These data excluded one subject who had a “virtually negligible” increase^54^. Clinically significant hyperkalemia has been reported with large supraphysiological doses in patients with renal failure (30g IV)^55^ and combined liver failure/renal impairment (again with very high dosing of 59-95g IV)^56^, in subjects taking spironolactone (an exclusion criterion in this study). Mean plasma K^+^ in 58 Indonesian subjects with uncomplicated malaria (in our initial NO studies in Jayapura) is at the low end of normal range (3.4 meql/L), with only 1 subject out of 58 having a K^+^> 4.0 [4.5 meq/L]. Subjects with renal failure or baseline K^+^≥4.2 meq/L will not be included in studies 2 and 3. Moreover lower doses will be used in both of our studies than the 30g dose used in published studies. Therefore a rise in K+ to ≥ 6.0 meq/L will be very unlikely.

Response: If serum K^+^ rises to ≥ 6.0 meq/L it will be immediately repeated using the bedside iSTAT to exclude hemolysis of sample. If K^+^ ≥ 6.0 meq/L or if significant ECG changes of hyperkalemia develop, the infusion will be stopped and subjects will be given 10ml of 10% calcium gluconate for membrane stabilization. This will be followed by continuous ECG monitoring and 10-15 minutely monitoring of K^+^: if thought necessary by the attending clinician, 10 U of regular insulin will be administered with 25g of IV glucose to induce K^+^ shift into cells.

- *Hypophosphatemia:*

A clinically insignificant transient fall in serum phosphate is noted with 30g infusions, with the maximum fall below baseline of 1.11 mg/100ml (or 33%), peaking 60 mins after commencement of a 30 min infusion^54^. In no cases did phosphate levels fall to potentially clinically significant levels (<1 mg/dL)^54^. We will nevertheless measure phosphate levels at baseline and at 60 mins.

Response: Moderate hypophosphatemia (1-2.5 mg/dL) is almost always asymptomatic and requires no therapy. Moreover, transient hypophosphatemia following arginine infusion *will not reflect any depletion of total body phosphate* that is required for very low levels of plasma phosphate to cause clinical manifestations. Low phosphate will therefore not be treated unless deemed essential by the treating clinician.

*c)* *Thrombophlebitis* is very unusual, but has been described^57^. This is minimized by using ≤10% solutions. One episode of *an extravasation reaction* has been described in a child. This will be minimized by administering arginine as a 10% solution, and careful insertion and observation of IV canula/symptoms/signs during infusion.

Response: If extravasation occurs, the infusion will be stopped, the affected extremity elevated. The catheter/needle will not be removed immediately, but rather left in place to aspirate fluid from the extravasated. The line will then be removed and an ice pack applied.

*d)* *Anaphylaxis*: We are aware of only one report of a case of *anaphylaxis^58^*. This would seem a very rare event with an exogenous/endogenous amino acid and is considered extremely unlikely in current studies

Response: The arginine infusion will be ceased. Adrenaline and rescucitation facilities will be readily available in the high care unit at RSMM, to which the clinical research ward is attached. Standard treatment for anaphylaxis will be undertaken in the very unlikely event it would be required.

*e)* *Rash:* maculopapular rash: rare; causality not proven. Response: infusion will be stopped

*f) Minor adverse effects:* Arginine can uncommonly cause nausea, vomiting, flushing, headache, and numbness. These will be documented in the CRF. These are also common in malaria, and will not result in cessation of the infusion, unless patient requests to withdraw from the study. In previous studies with oral arginine in SCD, gastrointestinal symptoms (including abdominal cramping and diarrhea) were occasionally seen (Claudia Morris, personal communication 2004), one of the reasons why they are now using IV arginine for the ongoing trial in SCD. Gastrointestinal side-effects (eg nausea, vomiting) have not been seen with IV infusion in SCD. Minor flushing has been seen.

**Theoretical Adverse Effects With No Documented Events**

*a) Effects on blood glucose:* arginine stimulates both insulin and glucagon release^53^. Because levels of glucagon increase to levels higher than that of insulin, the net effect in humans is reported to be a slight hyperglycemia^32,53^. Importantly, despite over 40 years’ of use of intravenous arginine in humans, there are **no** clinical case reports of hypoglycemia ever occurring after arginine infusion.

Hypoglycemia can occur in severe (but not, by definition, in uncomplicated malaria)^2^, therefore blood glucose will be rigorously monitored in both studies as outlined in table 2: We will use iSTAT cartridges (which use a few microlitres of blood) at the bedside to instantly measure glucose (as well as other electrolytes) prior to infusion, at the end of the 30 min infusion, and 15, 30, 60 and 210 minutes after the end of the infusion. Because levels of glucagon (which increases glucose) are higher after arginine infusion than insulin (which lowers glucose), if anything arginine may help prevent hypoglycemia in malaria. We will be measuring glucose in stage 2 and 3 in uncomplicated malaria, so can report back to the ethics committee the effect on blood glucose in malaria before we give arginine to anyone with severe malaria in future studies. If anything the glucagon effect *increasing* blood sugar is likely be protective.

Response: If blood glucose falls below 4 mmol/L, the arginine infusion will be stopped and 50% dextrose will be administered as per standard RSMM/MoH practice in severe malaria, with blood glucose continuing to be monitored 15 minutely to 60 mins then 30 minutely until 480 mins.

*b) Effect on pH:* Arginine hydrochloride contains H^+^ ions (4.75 mEq/g). While there is a *theoretical* potential for acidosis there have been no reports of this. Hertz *et al* (1972) ^56^ carefully documented the effect of IV arginine on arterial pH. They found **no change** in the pH despite infusion of 30g (much higher than we will use in this study) and despite the fact they infused arginine into patients with renal failure – who are at risk for acidosis^56^. Clinically significant change in pH has not been found except in patients with hypochloremic alkalosis (where arginine hydrochloride corrects the Cl deficiency and returns the alkalosis toward normal). We therefore do not expect any acidosis, but will monitor serial real-time venous pH, PCO_2_, chloride and bicarbonate at the bedside using iSTAT EC8+ cartridges.

Response: Arginine infusion will be ceased if venous bicarbonate falls below 15 mmol/L.

*c)* *Effect on kidney function:* We can find no case reports or primary clinical studies that report renal toxicity with L-arginine. In fact, two recent studies show that renal blood flow and glomerular filtration rate (the most accurate measure of renal function) **improve** after the infusion of L-arginine. Schlaich et al,^59^ studied patients with normal renal function and used an intravenous infusion of L-arginine at doses of 100mg/kg (7 Grams for a 70 kg adult) and 500mg/kg (35 grams for a 70 kg adult) over 30 minutes. They showed a statistically significant ***improvement*** in both renal blood flow and glomerular filtration rate at each dose. In the second study^60^, n intravenous dosage of 17mg/kg gave the same improvement in normal subjects. Reassuringly, in patients with renal impairment in this study, arginine infusion was safe and **did not** cause any worsening of renal blood flow or glomerular filtration rate. We therefore do not expect any change in renal function in studies 2 and 3, but we will monitored renal function by serial iSTAT bedside biochemistry (see Table 2).

*d) Consequences of tissue NO production from tissue NOS.* We believe that tissue NO production will have tissue-protective effects and will also reduce generation of superoxide from arginine-deficient NOS. Nevertheless we will be able to detect unexpected deleterious effects of increased tissue NO. As well as the organ-specific biochemical markers above (including creatinine, creatine kinase and liver enzymes/bilirubin), our existing lung function laboratory at RSMM and the exhaled NO apparatus will give us the unique opportunity to relate exhaled NO to measures of pulmonary gas transfer and alveolar dead space, sensitive markers of lung injury.

In the current sickle cell disease trial in the US children with acute vaso-occlusive disease, 36 patients (age 4-18 years) have been randomized to IV arginine 0.1 g/kg vs placebo. This has been very well tolerated, with no significant effect on blood pressure, liver function tests, electrolytes, glucose, renal function or vital signs (Claudia Morris, personal communication 2004). There have been no serious adverse events, and no acidosis

**Serious adverse events:** The following adverse events will be deemed serious, and will be notified immediately to the Chief Investigators and to the Chair of the Data Monitoring Committee for immediate review:

1. Death
2. Serum K^+^ rising > 6.0 meq/L with ECG changes
3. Anaphylaxis
4. SBP falling below 80 mmHg unresponsive to saline infusion
5. Clinical requirement for admission to High Care Unit, dialysis or ventilation

The doctor responsible for immediate management of adverse effects will be the internal medicine residents from Manado, currently Dr Denny. These are very experienced senior internal medicine residents.

**Limitations of Study 1 and 2:** Only patients with Hb≥6g/dl are included in this study. We do not believe that the PK-PD relationship will be significantly different in severe malarial anemia than in other forms of severe malaria, but cannot exclude this. Once the PK-PD relationship in severe malaria is known, in future studies we may be able to confirm its similarity in severe malaria anemia with fewer blood draws.

**Study 3:**

***Randomised concentration controlled trial, safety, and pharmacokinetic study in uncomplicated malaria:*** The pharmacokinetic-pharmacodynamic model [from studies 1 and 2)] will be used to determine an appropriate loading and maintenance dose of arginine in uncomplicated malaria, to achieve average plasma arginine concentrations of ~600 uM (a plasma level ~4 times the Km of CAT2 transporters for cellular uptake of extracellular arginine). *Prior to the commencement of study 3, the Data Monitoring Committee will review the dosing and sampling schedule we will calculate from the pharmacokinetic-pharmacodynamic model generated in studies 1-2.* In addition, the target concentration (600 mcM) may need to be readjusted based on the results from studies 1 and 2.

*Design:* Randomised Concentration Controlled Trial.

*Treatment allocation:* Patients with uncomplicated malaria will be randomized to receive one of two treatments:

i) Standard RSMM antimalarial drug regimen for falciparum malaria (currently quinine) plus normal saline placebo (loading dose of 100 ml over 30 mins plus maintenance infusion using same volume in [ii] below).

ii) Standard RSMM antimalarial drug regimen for falciparum malaria (currently quinine) plus IV arginine: loading dose as 30 min infusion (see CRF) followed by maintenance infusion over 12 hours. The doses of arginine will be determined from the pharmacokinetic-pharmacodynamic model to maintain a plasma level of 600uM.

An independent statistician will use computer-generated randomization to generate blocks of patient numbers for each treatment.

*Sample size:* Extrapolating from similar concentration controlled trials, we estimate approximately 20 subjects in each of the two groups. Sample size calculation will require the results of the natural history of arginine/NO/NOx levels during malaria (study 1), and the maximal improvement and time course of improvement following administration (study 2). Therefore the final sample size will be calculated based on the results of studies 1 and 2. The sample size will be reviewed by the Data Safety and Monitoring Committee, and each of the relevant ethics Committees will be notified prior to commencement of Study 3 with confirmation of the number of patients.

*Patient numbering and randomization*:

Once the patient has a confirmed diagnosis of falciparum malaria, has fulfilled all the inclusion criteria, has none of the exclusion criteria and has given written informed consent to participate, he/she will be allocated the next code of the study.

Randomization will be carried out in groups of 10, and each code given a sealed opaque envelope which will contain that patients’ treatment group and which will only be opened when a patient has been allocated a code number.

*Design:*

Cannula insertion and baseline sampling will be performed as in study 2. Serial vital signs, clinical assessments, and laboratory parameters of safety will be measured as in Study 2 above. As appropriate, we will use the model generated from studies 1 and 2, and modeling of any electrolyte disturbances to optimize the sampling schedule ***which will result in a reduced total number of pharmacokinetic and pharmacodynamic blood samples required compared with study 2***. The maximum total volume of blood drawn will again be ≤25ml, but because of reduced numbers of samples will likely be less. In addition we will measure preliminary biomarkers of efficacy: fever and parasite clearance times, serial lactate levels, plasma levels of endothelial adhesion molecules, biomarkers of inflammation and effects on physiological parameters (including vascular function, gas transfer and physiological deadspace), with frequency again guided by results from studies 1 and 2. The effect on NO production will be examined by serial measures of plasma NOx/creat, and exhaled NO, and effect on oxidant stress, including serial plasma glutathione (GSH):oxidised glutathione (GSSG) ratios (by HPLC)^61^.

Observations will continue for 24 hours after the completion of the arginine infusion, with the final assessment at this time being identical to the final assessment in study 2.

*Outcomes:*

In the arginine-treated group there will be global improvements in both biochemical (including plasma NOx/creatinine, exhaled NO) and physiological markers (including brachial artery dilatation, hyperemic blood flow on Doppler and physiological deadspace).

*Interpretation:* This data will generate more safety data in uncomplicated malaria. The data will be used to refine the linked pharmacokinetic-pharmacodynamic (pharmacokinetic-pharmacodynamic) model, with arginine levels being related to measures of NO production and oxidant stress as above. Determining if exogenous arginine results in an increment in NO production in malaria (± reduction in oxidant stress) and physiological parameters to be tested would be a major advance. It is hypothesised that arginine will result in improvements in clinically important outcomes in *severe malaria*. Because uncomplicated malaria is by definition a milder disease than severe malaria we do not necessarily expect to be able to detect improvements in clinically relevant outcomes in this study of uncomplicated malaria patients. We may be able to generate data on preliminary efficacy using biomarkers of outcome (eg lactate levels) in uncomplicated malaria, however lactate levels are usually only modestly elevated (or normal) in mild disease. An effect on lactate level will be sought but will not be required as part of the decision-making for proceeding to study 4 in severe malaria (where such an effect ***is*** expected).

*Feasibility:* RSMM has a clinical research unit attached to its intensive care unit. As part of our ongoing severe malaria studies, RSMM-MSHR-NIHRD research staff are experienced in assessing patients for fulfilment of modified WHO criteria for severe malaria and ensuring consent is informed by a thorough explanation of the study. The laboratory at RSMM has a dedicated research microscopist/technician, -70ºC freezer, refrigerated centrifuges and biosafety cabinet, and the bedside iSTAT apparatus can rapidly assay biochemical measures of safety. Supervision and quality assurance of exhaled NO, vascular and lung physiology will be undertaken by experienced collaborators. Our team members in Timika are fully familiar with lung function tests as part of our current lung-malaria studies. Pharmacokinetic-pharmacodynamic modeling, and protocol design for study 3 will be done under the supervision of an experienced pharmacometrician (Dr Duffull), statistician (Dr Simpson) and Dr Retno (NIHRD).

*Proposed timeline (subject to ethics approval process):*

October 2004: planning visit, finalisation of logistics, setting up exhaled NO apparatus, deadspace equipment and troubleshooting equipment.

Dec 2004- Nov 2005: study 1 *(subject to ethics approval process):*

Dec 2004 -April 2005: Study 2

Feb 2005- Oct 2005: measurements of arginine, and NOx:creat

April-August 2005: PD-PD modelling. Design of study 3: review by Data Safety Monitoring Committee

Dec 2005- April 2006: Study 3.

Nov 2005- mid 2006: measurements of arginine, and NOx:creat; further PK-PD modelling. Design of Study 4 (severe malaria) to be submitted to ethics for review.

*Ethics*

Studies 1-3 above will be reviewed by the Ethics Committee of National Institute of Health Research, Ministry of Health, Jakarta, Indonesia and the Ethics Committee of Menzies School of Health, Darwin, Australia. The data generated in studies 1-3 will be used to decide whether proceeding to a study in *severe malaria* is warranted, and if so, to design an appropriate protocol for undertaking the safety and pharmacokinetic-pharmacodynamic study in *severe* malaria. The future severe malaria protocol will be submitted to each of the Ethics Committees as a separate future submission based on the results from studies 1-3.

***Ethical issues:***

*Potential side effects of arginine:* Patients with uncomplicated malaria have mean levels of arginine *below* normal, and combined with a dose well below the standard 30g IV dose used in other settings, we do not expect significant adverse effects to occur. Whilst we believe adverse effects to be unlikely, our protocol outlined in “study 2” above allows us to detect, and if necessary rectify them:

*Standard treatment:* Regardless of whether they receive arginine, all patients will receive prompt standard antimalarial drug treatment based on standard Depkes and RSMM protocols, currently quinine. There will be no delay in starting treatment due to participation in this study.

*Blood samples:* the maximum total of 25ml of blood taken over 21-72 hours is safe. Those with significant anemia (Hb <6 g/dL) will be excluded. Serial sampling is essential for appropriate pharmacokinetic-pharmacodynamic modeling. However serial blood sampling has the potential to cause concern to patients. In northern Australia, we have successfully used flip charts during the consent process to illustrate visually the procedure of studies like this that involve blood sampling. We will use similar flip charts to demonstrate visually the total volume of blood that will be collected. The flip chart will illustrate that the very small volumes collected during serial bleeds add up to only a relatively small and safe volume of blood. The use of these flip charts have proved to be especially useful in explaining blood volumes in patients with limited education who have limited numeracy and literacy skills.

*Cannula:* The consent process will explain how the cannula will be the same as that used clinically for medications and fluids. The information process will clarify that the cannula will ensure that the serial samplings will be painless and avoid the need for further painful needlesticks or fingerpricks. We will also explain that the blood taken from the cannula will also be used for any serial clinical tests requested by the treating RSMM doctor (eg repeat parasite counts, blood glucose, repeat hemoglobin), thus avoiding painful followup fingerpricks required for clinical purposes. Moreover, the serial iSTAT readings will be useful to the treating doctor, who will be alerted to abnormal results of clinical importance.

*Consent:* The Written Subject Information Statement will be translated into Bahasa Indonesia following any revisions recommended by the NIHRD ethics committees. If Bahasa Indonesia is a second language, the information will be translated with a bilingual RSMM staff member.

*Confidentiality:* Results will be stored electronically at RSMM and later at MSHR/NIHRD, accessible to investigators only, and password protected. Data sheets will be stored in a locked, secure location at RSMM.

*Non-invasive vascular studies:* These *forearm* (*not* above elbow) measures are not painful. The subject may experience minor discomfort during inflation of pressure cuffs during the studies. This discomfort will be transient and is no worse than having blood pressure measurement in routine clinical practice.

*Lung function studies:* The pulmonary function tests to be used in this study are non-invasive and routinely used in clinical, non-research pulmonary practice in hospitals throughout the world and have been shown to be safe and reliable, even in patients with severe lung disease. The tests do not involve radiation or other risk. They have been well tolerated in our malaria studies to date

*Why not start with studies with oral arginine?*

The known variability in bioavailability of oral arginine will make it much more difficult to model the pharmacodynamic effects of arginine. Oral arginine studies in malaria will require many more dosing levels, and many more subjects at each dosing level, and potentially a greater number of blood draws. We would thus need to enroll many more patients than we currently propose. We do not believe it is ethical to significantly increase the numbers of subjects we enroll to obtain less-interpretable data, when a smaller number of carefully studied subjects given IV arginine will give better quality information. Peer-review of our proposal before submission to the Wellcome Trust was that we would be criticized scientifically for starting with oral administration for the reasons above. The greater number of subjects and greater numbers of samplings involved will increase the invasiveness involved for subjects with uncomplicated malaria. IV therapy is safe. Excluding the confounding effect of variable bioavailability by using IV administration will mean that we obtain the maximal amount of high quality data from the minimum number of subjects, and minimum number of dosing levels and samplings. Overall this will make the study ***less invasive*** than if we use oral administration.

Moreover, we need to demonstrate **proof of principle** in malaria: that a known dose of arginine is able to increase NO production or have a clinically important measurable effect on the outcomes of interest. If we start with oral arginine studies and if absorption is poor in malaria (or transport processes altered in malaria), we may discard a potentially valuable adjunctive treatment by failing to demonstrate a significant effect. If known amounts of IV arginine do not increase blood arginine levels in malaria, cannot increase NO production or do not have a measurable effect on the outcomes of interest, then the studies should stop and oral bioavailability studies will be unnecessary.

In subjects seriously unwell with severe malaria, it is likely that IV therapy will be required for any adjunctive therapy, whether it is arginine or any other potential adjunctive therapy. IV administration is our target route for administration in severe malaria. If IV arginine is effective in severe malaria, enteral/nasogastric arginine *may then* be feasible, and appropriate to study.

*Are we using a new experimental drug?* Arginine has been administered to humans orally and intravenously for decades for diagnostic and therapeutic use, with extensive safety data. It is commonly used diagnostically in humans as a 30g intravenous dose to stimulate growth hormone release. Arginine is therefore not a new experimental drug. We are simply examining whether an established natural product/drug can be used for a ***new indication*** (ie as an adjunctive treatment for malaria combined with standard antimalarial drug).

*Why not use an animal model?* No animal model of malaria is an exact model for human disease and all have limitations. Most importantly, in none of the current animal models is NO production reduced in malaria. This is in direct contrast to what happens in humans, where NO production is reduced, and where reduced NO production and low arginine levels are associated with poor outcome. The international reviewers for this proposal agreed that it would waste time, resources and animals to test arginine in animal models of malaria, because results would not be applicable to humans.

*Funding:* Funding is from the Wellcome Trust (a non-profit UK-based medical charity) and the National Health and Medical Research Council (Australia) as part of the International Collaborative Research Grants Scheme awarded jointly to NIHRD and MSHR. *No funding has or will be received from any companies manufacturing arginine.* Arginine is prepared as an inexpensive off-patent generic commercially available drug (see generic product information attached).

## Authorship/ownership of the study: will be shared by all investigators and, as appropriate, those providing significant consultant support, as per International Committee of Medical Journal Editors 2003 criteria.

*Certificate of analysis for intraveneous arginine:* This will provided to Indonesian FDA for approval following ethical review.

| **TABLE 1**  **(stage 1)**  Time (mins) | Clinic sample  (Day 0) | 0 | 60-240 | 480-720 | Day 1 | Day 1 | Day 2  § | Day 3 §§  (severe malaria only) | 1-2 week followup |
| --- | --- | --- | --- | --- | --- | --- | --- | --- | --- |
| Time (hrs) | -5 to -1 | 0 | 1-4 | 8-12 | 18-24 | 30-36 | 42-48 | ±72 | 1-2 wks |
| Volume of blood (mL) |  | 10 |  | 2 (UM only) | 2 | 2 | 2 | 2 (SM only) | 2 |
| Vital signs (BP, PR, RR) 2nd hourly for 4 hours, then 2-4 hourly |  | X | X | X | X | X | X |  | X |
| Clinical symptoms/signs 4 hourly |  | X | X | X | X | X | X | X | X |
| iSTAT EC8+ #* or electrolytes on cryopreserved plasma** | X | X | X | X | X |  | X | X |  |
| Phosphate** |  | X | X | X | X |  | X | X |  |
| Thick/thin film* | X | X | X | X |  |  | X | X | X |
| Lactate (CG4+)* |  | X | X |  | X |  | X § | X |  |
| LFTs, CK, Creatinine** |  | X |  | X | X |  | X | X |  |
| Exhaled NO* |  | X | X | X | X | X | X | X | X |
| Gas transfer (DLCO)* |  | X | X |  | X |  | X | X | X |
| Physiological dead space* |  | X | X |  | X |  | X | X |  |
| Endothelial function testing* |  | X | X |  | X |  | X | X | X |
| Amino acids (including argine), NOx, oxidant markers** |  | X | X | X | X | X | X | X | X |
| Urine (pterins, NOx)** |  |  | X*** | X | X | X | X*** | X | X |

| **TABLE 2 (stage 2)**  Time (mins) | Clinic sample | -60 | -45 | -30 | -15 | 0 | 30 | 45 | 60 | 90 | 180 | 480 | Next AM | 1-2 weeks |
| --- | --- | --- | --- | --- | --- | --- | --- | --- | --- | --- | --- | --- | --- | --- |
| Time (hrs) | -5 to -2 | -1 | -3/4 | - ½ | - ¼ | 0 | ½ | ¾ | 1 | 1 ½ | 3 | 8 | 18-24 | 1-2 weeks |
| Volume of blood (mL) |  | 7 |  |  |  | 2 | 2 | 2 | 2 | 2 | 2 | 2 | 2 | 2 |
| Vital signs (BP, PR, RR) 10-15 minutely |  | X | X | X | X |  |  |  | X | X |  |  |  |  |
| Vital signs 5-10 minutely |  |  |  |  |  | X | X | X |  |  |  |  |  |  |
| Vital signs 30 minutely |  |  |  |  |  |  |  |  |  |  | X |  |  |  |
| Vital signs 2-4 hourly |  |  |  |  |  |  |  |  |  |  |  | X | X | X |
| Clinical symptoms/signs‡ |  | X |  |  |  | X | X | X | X | X | X | X | X | X |
| iSTAT EC8+ #* |  | X |  |  |  | X | X | X | X | X | X | X | X |  |
| Phosphate ** |  | X |  |  |  |  | X | X | X | X | X | X | X |  |
| Thick/thin film* | X | X |  |  |  |  |  |  |  |  | X | X | X | X |
| Lactate at RSMM* |  | X |  |  |  |  |  |  |  |  | X |  | X |  |
| LFTs, CK, Creatinine** |  | X |  |  |  |  |  |  |  |  |  | X | X | X |
| Exhaled NO* |  | X |  |  |  | X | X | X | X | X | X | X | X | X |
| Gas transfer (DLCO)* |  |  |  | X |  |  |  |  | X |  |  |  | X | X |
| Physiological dead space* |  | X |  |  |  |  |  |  | X |  |  |  | X |  |
| Endothelial function testing* |  |  | X |  |  |  |  | X |  |  |  |  | X | X |
| Amino acids (incl. arginine), NOx, ICAM, oxidant markers** | X | X |  |  |  | X | X | X | X | X | X | X | X | X |
| Urine (pterins, NOx)** |  |  |  | X |  |  |  |  |  |  | X*** |  | X*** | X |

**Footnotes for tables 1 and 2:**

Arginine infusion (Table 2): from time 0-30 mins

# iSTAT EC8+ is a bedside biochemistry cartridge which uses only several microlitres to measure Na^+^, K^+^, Cl^-^, pH, PCO_2_, urea nitrogen (BUN)/urea, glucose, hematocrit, TCO_2_, bicarbonate, Base excess, anion gap, hemoglobin.

ECG will be monitored continuously for up to 6 hours after arginine infusion and repeated at 20 hours. Temperature will also be monitored 4-6 hourly until 20 hours.

‡ Clinical symptom and sign assessment: questioned on presence of headache, lightheadedness, nausea, vomiting, flushing, numbness, difficulty breathing, pain at infusion site; and examined for rash.

Location of each testing procedure: A strength of this study is that all the endothelial physiology measurements, all the lung physiology measurements, all the parasite counts, the lactate and phosphate measurements, and most of the other biochemical measurements (eg iSTAT bedside biochemistry) will be undertaken **on site** at RSMM in Timika (either at the bedside or at the RSMM lab), with on-the-job training of all local and visiting staff involved. The asterisks (* or **) identify which measurements will be done at RSMM in Timika (*) and which at MSHR (**) or University of Sydney (oxidant stress measurements).

*performed at RSMM.

** performed in Darwin

*** Urine will be collected at approx 4 hours, and at each time voided thereafter.

§ if patient still in hospital

§§ in severe malaria patients only

**References**

1. Guerin PJ, Olliaro P, Nosten F, et al. Malaria: current status of control, diagnosis, treatment, and a proposed agenda for research and development. *Lancet Infect Dis* 2002;**2**(9)**:**564-73.

2. WHO. Severe falciparum malaria. *Trans R Soc Trop Med Hyg* 2000;**94, suppl 1:**S1-S90.

3. Moncada S, Higgs A. The L-arginine-nitric oxide pathway. *New England Journal of Medicine* 1993;**329:**2002-2012.

4. Weinberg J. Nitric oxide production and nitric oxide synthase type 2 expression by human mononuclear phagocytes: a review. *Molec Med* 1998;**4:**557-591.

5. Degroote MA, Fang FC. NO inhibitions. Antimicrobial properties of nitric oxide. *Clinical Infectious Diseases* 1995;**21**(Suppl 2)**:**S 162-S 165.

6. Anstey N, Weinberg J, Granger D. Nitric oxide and malaria. In: Fang F, ed. Nitric oxide and infection. New York: Plenum Publishing Corp, 1999: 311-341.

7. Wu G, Morris S. Arginine metabolism: nitric oxide and beyond. *Biochem J* 1998;**336:**1-17.

8. Xia Y, Roman LJ, Masters BS, Zweier JL. Inducible nitric-oxide synthase generates superoxide from the reductase domain. *J Biol Chem* 1998;**273**(35)**:**22635-9.

9. Vasquez-Vivar J, Kalyanaraman B, Martasek P, et al. Superoxide generation by endothelial nitric oxide synthase: the influence of cofactors. *Proc Natl Acad Sci U S A* 1998;**95**(16)**:**9220-5.

10. Wang W, Wang S, Yan L, et al. Superoxide production and reactive oxygen species signaling by endothelial nitric-oxide synthase. *J Biol Chem* 2000;**275**(22)**:**16899-903.

11. Morris CR, Kuypers FA, Larkin S, Vichinsky EP, Styles LA. Patterns of arginine and nitric oxide in patients with sickle cell disease with vaso-occlusive crisis and acute chest syndrome. *J Pediatr Hematol Oncol* 2000;**22**(6)**:**515-20.

12. Morris CR, Kuypers FA, Larkin S, et al. Arginine therapy: a novel strategy to induce nitric oxide production in sickle cell disease. *Br J Haematol* 2000;**111**(2)**:**498-500.

13. Yu YM, Ryan CM, Castillo L, et al. Arginine and ornithine kinetics in severely burned patients: increased rate of arginine disposal. *Am J Physiol Endocrinol Metab* 2001;**280**(3)**:**E509-17.

14. Freund H, Atamian S, Holroyde J, Fischer JE. Plasma amino acids as predictors of the severity and outcome of sepsis. *Ann Surg* 1979;**190**(5)**:**571-6.

15. Argaman Z, Young V, Noviski N, et al. Arginine and nitric oxide metabolism in critically ill septic pediatric patients. *Crit Care Med* 2003;**31**(2)**:**February: in press.

16. Boger RH, Bode-Boger SM. The clinical pharmacology of L-arginine. *Annu Rev Pharmacol Toxicol* 2001;**41:**79-99.

17. Bode-Boger SM, Boger RH, Galland A, Tsikas D, Frolich JC. L-arginine-induced vasodilation in healthy humans: pharmacokinetic-pharmacodynamic relationship. *Br J Clin Pharmacol* 1998;**46**(5)**:**489-97.

18. Anstey NM, Weinberg JB, Hassanali MY, et al. Nitric oxide in Tanzanian children with malaria: inverse relationship between malaria severity and nitric oxide production/nitric oxide synthase type 2 expression. *J Exp Med* 1996;**184**(2)**:**557-67.

19. Perkins DJ, Kremsner PG, Schmid D, Misukonis MA, Kelly MA, Weinberg JB. Blood mononuclear cell nitric oxide production and plasma cytokine levels in healthy gabonese children with prior mild or severe malaria. *Infect Immun* 1999;**67**(9)**:**4977-81.

20. Hobbs MR, Udhayakumar V, Levesque MC, et al. A new NOS2 promoter polymorphism associated with increased nitric oxide production and protection from severe malaria in Tanzanian and Kenyan children. *Lancet* 2002;**360**(9344)**:**1468-75.

21. Anstey NM, Boutlis CS, Tjitra E, et al. Inverse association between disease severity and systemic nitric oxide (NO) production and mononuclear cell inducible NO synthetase (NOS2) expression in Indonesian adults with malaria suggests anti-disease effects of NO. *Am J Trop Med Hyg* 2001;**65**(3)**:**Suppl.: 400.

22. Florquin S, Amraoui Z, Dubois C, Decuyper J, Goldman M. The protective role of endogenously synthesized nitric oxide in staphylococcal enterotoxin B-induced shock in mice. *J Exp Med* 1994;**180:**1153-1158.

23. De Caterina R, Libby P, Peng HB, et al. Nitric oxide decreases cytokine-induced endothelial activation. Nitric oxide selectively reduces endothelial expression of adhesion molecules and proinflammatory cytokines. *J Clin Invest* 1995;**96**(1)**:**60-8.

24. Maneerat Y, Viriyavejakul P, Punpoowong B, et al. Inducible nitric oxide synthase expression is increased in the brain in fatal cerebral malaria [In Process Citation]. *Histopathology* 2000;**37**(3)**:**269-77.

25. Lopansri B, Anstey N, Weinberg J, et al. Low plasma arginine concentrations in children with cerebral malaria and decreased nitric oxide production. *Lancet* 2003;**361:**February: in press.

26. Vincendeau P, Gobert AP, Daulouede S, Moynet D, Djavad Mossalayi M. Arginases in parasitic diseases. *Trends Parasitol* 2003;**19**(1)**:**9-12.

27. Das BS, Mohanty S, Mishra SK, et al. Increased cerebrospinal fluid protein and lipid peroxidation products in patients with cerebral malaria. *Trans R Soc Trop Med Hyg* 1991;**85**(6)**:**733-4.

28. Das BS, Patnaik JK, Mohanty S, et al. Plasma antioxidants and lipid peroxidation products in falciparum malaria. *Am J Trop Med Hyg* 1993;**49**(6)**:**720-5.

29. Jones SM, Thurman RG. L-arginine minimizes reperfusion injury in a low-flow, reflow model of liver perfusion. *Hepatology* 1996;**24**(1)**:**163-8.

30. Gyan B, Troye-Blomberg M, Perlmann P, Bjorkman A. Human monocytes cultured with and without interferon-gamma inhibit *Plasmodium falciparum* parasite growth in vitro via secretion of reactive nitrogen intermediates. *Parasite Immunol* 1994;**16**(7)**:**371-5.

31. Seguin MC, Klotz FW, Schneider I, et al. Induction of nitric oxide synthase protects against malaria in mice exposed to irradiated Plasmodium berghei infected mosquitoes: involvement of interferon gamma and CD8+ T cells. *J Exp Med* 1994;**180**(1)**:**353-8.

32. Barbul A. Arginine: biochemistry, physiology, and therapeutic implications. *JPEN J Parenter Enteral Nutr* 1986;**10**(2)**:**227-38.

33. Ceremuzynski L, Chamiec T, Herbaczynska-Cedro K. Effect of supplemental oral L-arginine on exercise capacity in patients with stable angina pectoris. *Am J Cardiol* 1997;**80**(3)**:**331-3.

34. Lerman A, Burnett JC, Jr., Higano ST, McKinley LJ, Holmes DR, Jr. Long-term L-arginine supplementation improves small-vessel coronary endothelial function in humans. *Circulation* 1998;**97**(21)**:**2123-8.

35. Grasemann H, Gartig SS, Wiesemann HG, Teschler H, Konietzko N, Ratjen F. Effect of L-arginine infusion on airway NO in cystic fibrosis and primary ciliary dyskinesia syndrome. *Eur Respir J* 1999;**13**(1)**:**114-8.

35a. Lorente JA et al, L-arginine pathway in sepsis syndrome. Crit Care Med 1993; 21: 1287-1295

36. Boger R, SM B-B. The clinical pharmacology of L-arginine. *Annu Rev Pharmacol Toxicol* 2001;**41:**79-99.

37. Beale RJ, Bryg DJ, Bihari DJ. Immunonutrition in the critically ill: a systematic review of clinical outcome. *Crit Care Med* 1999;**27**(12)**:**2799-805.

38. Tepaske R, Velthuis H, Oudemans-van Straaten HM, et al. Effect of preoperative oral immune-enhancing nutritional supplement on patients at high risk of infection after cardiac surgery: a randomised placebo-controlled trial. *Lancet* 2001;**358**(9283)**:**696-701.

39. Barbul A. Immunonutrition comes of age. *Crit Care Med* 2000;**28**(3)**:**884-5.

40. Traber DL. Arginine in shock, trauma, and sepsis. *Crit Care Med* 2002;**30**(3)**:**705-6.

41. Galban C, Montejo JC, Mesejo A, et al. An immune-enhancing enteral diet reduces mortality rate and episodes of bacteremia in septic intensive care unit patients. *Crit Care Med* 2000;**28**(3)**:**643-8.

41a Schon T et al. Arginine as an adjunct to chemotherapy improves clinical outcome in active tuberculosis. Eur Resp J 2003; 21: 483-488

42. Tran TH, Day NP, Nguyen HP, et al. A controlled trial of artemether or quinine in Vietnamese adults with severe falciparum malaria. *N Engl J Med* 1996;**335**(2)**:**76-83.

43. Hyland K, Howells DW. Analysis and clinical significance of pterins. *J Chromatogr* 1988;**429:**95-121.

44. Recommendations for standardized procedures for the on-line and off-line measurement of exhaled lower respiratory nitric oxide and nasal nitric oxide in adults and children-1999. This official statement of the American Thoracic Society was adopted by the ATS Board of Directors, July 1999. *Am J Respir Crit Care Med* 1999;**160**(6)**:**2104-17.

45. Celermajer DS, Adams MR, Clarkson P, et al. Passive smoking and impaired endothelium-dependent arterial dilatation in healthy young adults. *N Engl J Med* 1996;**334**(3)**:**150-4.

46. Celermajer DS, Sorensen KE, Gooch VM, et al. Non-invasive detection of endothelial dysfunction in children and adults at risk of atherosclerosis. *Lancet* 1992;**340**(8828)**:**1111-5.

47. Griffiths KA, Sader MA, Skilton MR, Harmer JA, Celermajer DS. Effects of raloxifene on endothelium-dependent dilation, lipoproteins, and markers of vascular function in postmenopausal women with coronary artery disease. *J Am Coll Cardiol* 2003;**42**(4)**:**698-704.

48. Raitakari OT, Celermajer DS. Flow-mediated dilatation. *Br J Clin Pharmacol* 2000;**50**(5)**:**397-404.

49. Turner GD, Morrison H, Jones M, et al. An immunohistochemical study of the pathology of fatal malaria. Evidence for widespread endothelial activation and a potential role for intercellular adhesion molecule-1 in cerebral sequestration. *Am J Pathol* 1994;**145**(5)**:**1057-69.

50. Serirom S, Raharjo WH, Chotivanich K, Loareesuwan S, Kubes P, Ho M. Anti-adhesive effect of nitric oxide on Plasmodium falciparum cytoadherence under flow. *Am J Pathol* 2003;**162**(5)**:**1651-60.

51. Beal SL, Sheiner L. NONMEM User's Guide, parts I-VII. 1992**:**San Francisco: NONMEM Project Group.

52. Annane D, Sanquer S, Sebille V, et al. Compartmentalised inducible nitric-oxide synthase activity in septic shock. *Lancet* 2000;**355**(9210)**:**1143-8.

53. Palmer JP, Walter RM, Ensinck JW. Arginine-stimulated acute phase of insulin and glucagon secretion. I. in normal man. *Diabetes* 1975;**24**(8)**:**735-40.

54. Massara F, Martelli S, Cagliero E, Camanni F, Molinatti GM. The hypophosphatemic and hyperkalemic effect of arginine in man. *J Endocrinol Invest* 1980;**3**(2)**:**177-80.

55. Bushinsky DA, Gennari FJ. Life-threatening hyperkalemia induced by arginine. *Ann Intern Med* 1978;**89**(5 Pt 1)**:**632-4.

56. Hertz P, Richardson JA. Arginine-induced hyperkalemia in renal failure patients. *Arch Intern Med* 1972;**130**(5)**:**778-80.

57. Baker GL, Franklin JD. Management of arginine monohydrochloride extravasation in the forearm. *South Med J* 1991;**84**(3)**:**381-4.

58. Tiwary CM, Rosenbloom AL, Julius RL. Anaphylactic reaction to arginine infusion. *N Engl J Med* 1973;**288**(4)**:**218.

59. Schlaich MP, Jacobi J, John S, Delles C, Fleischmann I, Schmieder RE. Is l-arginine infusion an adequate tool to assess endothelium-dependent vasodilation of the human renal vasculature? *Clin Sci (Lond)* 2000;**99**(4)**:**293-302.

60. Koller-Strametz J, Wolzt M, Fuchs C, et al. Renal hemodynamic effects of L-arginine and sodium nitroprusside in heart transplant recipients. *Kidney Int* 1999;**55**(5)**:**1871-7.

61. Jones DP. Redox potential of GSH/GSSG couple: assay and biological significance. *Methods Enzymol* 2002;**348:**93-112.

**INFORMATION SHEET**

**“Arginine in malaria: study 1”**

Malaria is a common infection in Indonesia and may cause serious illness. This form tells you about a research project that is looking at whether natural substances in the blood called arginine and nitric oxide protect people against malaria and what happens to them as people recover from malaria.

This study will see how quickly levels of arginine come back to normal as you get better from malaria and whether this is related to the amount of nitric oxide you make. We need to know this so that we can plan how much arginine we may need to give people in future if they get a bad attack of malaria.

If you agree to join this research study we will use a small needle to put a thin plastic tube into one of your veins. This is momentarily painful. We will use this to take a small amount of blood (10 ml or 2 teaspoons). Some of this blood will be used to treat and monitor your infection, and some of the blood will be used for malaria research. It will be examined for arginine, nitric oxide, and substances related to your malaria infection. We will leave this cannula in your vein for 1-3 days while you are being treated for malaria, and use this to take smaller amounts of blood up to five more times over 1-3 days. This hurt means you will not have to have repeated painful fingerpricks or needles for taking blood: these blood samples will be used by your doctor to treat and monitor your infection and some will be used for malaria research. The total amount of blood we will take will be no more than 25 mls (or 5 teaspoons) which is a safe amount.

We will also ask to obtain a small amount of your urine now and whenever you need to pass urine. We will also ask you to breathe into a tube every few hours to check the amount of nitric oxide in your breath and how well your lungs work. These are tests used routinely in hospitals to check peoples’ lungs, and do not hurt.

We will also use an ultrasound test to measure the size of one of your blood vessels at the elbow plus blood flow within it. This technique is safe and painless, and does not cause any harm. The scan of your arm will be done with you resting, lying down and relaxing. We will then put a blood pressure cuff on your forearm and inflate the cuff for 4 minutes. When the cuff is let down, we will do a second scan. Inflating the cuff will be uncomfortable, like having your blood pressure recorded, but does not cause damage. Then there will be a break for 10-15 minutes. We will then do another scan with you resting and relaxing. We will repeat these ultrasound tests a few hours later and again each day while you are in hospital.

This is research to try to understand how people recover from malaria. You will receive the standard treatment for malaria recommended by the Ministry of Health of the Republic of Indonesia. Your participation in the research will not cost you or your family any money. If your doctor may recommend some other tests and procedures to better diagnose and treat your infection. If you decide not to participate in the study, you will still receive this standard treatment and care for malaria. You may withdraw from the study at any time and still receive standard treatment and care.

There will be no name or other patient identification in any study report which will be published later on. You may contact Dr Rini on 901-301 827 at any time to answer any questions about the study. If you have any concerns about your rights or the conduct of the study you may contact Dr Liliana Kurniawan on 21-425 9860 or Linda Ward in Australia on 61-8-8922 7922.

Benefits of the study: if you have malaria you will receive the usual treatment for malaria and will not otherwise receive direct benefit from the study. However by understanding how quickly arginine levels recover during malaria, we hope to know how much arginine to give people with malaria in future.

**CONSENT TO PARTICIPATE IN A MALARIA RESEARCH STUDY**

**“Arginine in malaria: study 1”**

**This form means I can say “No”**

I have read and understood the information sheet attached, and have been given the opportunity to discuss it and ask questions. I agree that my samples can be used in the research outlined on the information sheet. I understand that I do not have to participate in this study. If I do not participate in the study I will still receive the usual treatment.

I also DO / DO NOT agree for my samples to be stored indefinitely for potential future use for research related to people respond to or are protected from malaria. I understand that my stored samples will not be used for any other purpose.

By my signature below I consent to participate:

___________________________ ______________________________ __________

Name of study subject Signature of study subject/next of kin Date

_________________________________ ______________________________ __________

Name of person obtaining consent Signature of person obtaining consent Date

**INFORMATION SHEET: “Arginine in malaria: study 2”**

Malaria is a common infection in Indonesia and may cause serious illness. This form tells you about a research project that is looking at whether a natural substance produced by the body called arginine can be given as an extra treatment to help people get better from malaria.

In the future we want to be able to give extra arginine to people with bad malaria to try to stop them from dying. In order to know how much to give we are doing this study in people like you with a mild form of malaria. This study will involve giving you some arginine treatment, plus the standard treatment for malaria recommended by the Ministry of Health of the Republic of Indonesia.

If you agree to join this research study we will use a small needle to put a thin plastic tube (cannula) into a vein in each of your arms. This is momentarily painful and there is a small risk of bleeding or bruising. We will use one of the cannulas to take a small amount of blood. Some of this blood will be used to treat and monitor your infection, and some of the blood will be used for malaria research. It will be examined for arginine, nitric oxide, and substances related to your malaria infection. We will leave this cannula in your vein overnight while you are being treated for malaria, and use this to take smaller amounts of blood up to 9 more times over about 20 hours. When we take blood from the cannula it does not hurt and you will not have to have repeated painful fingerpricks or needles for taking blood: these blood samples will be used by your doctor to treat and monitor your infection and some will be used for malaria research. The total amount of blood we will take will be no more than 25 mls (or 5 teaspoons) which is a safe amount.

We will also measure your pulse, blood pressure and heart tracing and ask you questions every few minutes when we give the arginine, and then less frequently after that. We will ask to obtain a small amount of your urine now and whenever you need to pass urine. We will also ask you to breath into a tube every 30 minutes at first and later every few hours to check the amount of nitric oxide in your breath and how well your lungs work. These are tests used routinely in hospitals to check peoples’ lungs, and do not hurt.

We will also use an ultrasound test to measure the size of one of your blood vessels at the elbow plus blood flow within it. This technique is safe and painless, and does not cause any harm. The scan of your arm will be done with you resting, lying down and relaxing. We will then put a blood pressure cuff on your forearm and inflate the cuff for 4 minutes. When the cuff is let down, we will do a second scan. Inflating the cuff will be uncomfortable, like having your blood pressure recorded, but does not cause damage. Then there will be a break for 10-15 minutes. We will then do another scan with you resting and relaxing. We will repeat these ultrasound tests up to two times, with the last time being tomorrow morning.

Your participation in the research will not cost you or your family any money. Your doctor may recommend some other tests and procedures to better diagnose and treat your infection. If you decide not to participate in the study, you will still receive this standard treatment and care for malaria. You may withdraw from the study at any time and still receive standard treatment and care. There will be no name or other ident-ification in any study report which will be published later on. You may contact Dr Rini on 901-301 827 at any time to answer any questions about the study. If you have any concerns about your rights or the conduct of the study you may contact Dr Liliana Kurniawan on 21-425 9860 or Linda Ward on +61-8-8922 7922.

***Benefits of the study:*** People who have a milder form of malaria like you usually get better quickly with the usual treatment for malaria and may or may not not receive direct benefit from the extra arginine treatment study used in this study. However by understanding how much arginine we need to give to people with malaria, we hope to be able to give this extra treatment to people with bad malaria in the future.

***Risks from the study:*** Arginine is a natural substance in the body and in many foods, but is also available as an injection in hospitals. It has been used as a standard drug in hospitals for many years in larger doses than we will use today. Even when used in bigger doses than we will use it is usually very safe. Sometimes large doses can cause the blood pressure to go down and sometimes people may feel dizzy, feel sick, headache or flushing. Other times it can cause some of the salts in the blood to go up or down but it is very rare for this to cause problems. It can also cause irritation of the veins or very rarely an allergic reaction. Because we will use smaller doses, we do not expect these to be a problem for you. We will watch for any side-effects and can give you treatment if any of them develop.

**CONSENT TO PARTICIPATE IN A MALARIA RESEARCH STUDY**

**“Arginine in malaria: study 2”**

**This form means I can say “No”**

I have read and understood the information sheet attached, and have been given the opportunity to discuss it and ask questions. I agree that my samples can be used in the research outlined on the information sheet. I understand that I do not have to participate in this study. If I do not participate in the study I will still receive the usual treatment.

I also DO / DO NOT agree for my samples to be stored indefinitely for potential future use for research related to people respond to or are protected from malaria. I understand that my stored samples will not be used for any other purpose.

By my signature below I consent to participate:

___________________________ ______________________________ _____________

Name of study subject Signature of study subject/next of kin Date

_________________________________ ______________________________ _____________

Name of person obtaining consent Signature of person obtaining consent Date

**INFORMATION SHEET: “Arginine in malaria: study 3”**

Malaria is a common infection in Indonesia and may cause serious illness. This form tells you about a research project that is looking at whether a natural substance produced by the body called arginine can be given as an extra treatment to help people get better from malaria.

In the future we want to be able to give extra arginine to people with bad malaria to try to stop them from dying. In order to know how much to give we are doing this study in people like you with a mild form of malaria. This study will involve giving you the standard treatment for malaria recommended by the Ministry of Health plus, either arginine treatment or placebo treatment.

If you agree to join this research study we will use a small needle to put a thin plastic tube (cannula) into a vein in each of your arms. This is momentarily painful and there is a small risk of bleeding or bruising. We will use one of the cannulas to take a small amount of blood. Some of this blood will be used to treat and monitor your infection, and some of the blood will be used for malaria research. It will be examined for arginine, nitric oxide, and substances related to your malaria infection. We will leave this cannula in your vein overnight while you are being treated for malaria, and use this to take smaller amounts of blood up to 9 more times over about 20 hours. When we take blood from the cannula it does not hurt and you will not have to have repeated painful fingerpricks or needles for taking blood: these blood samples will be used by your doctor to treat and monitor your infection and some will be used for malaria research. The total amount of blood we will take will be no more than 25 mls (or 5 teaspoons) which is a safe amount.

We will also measure your pulse, blood pressure and heart tracing and ask you questions every few minutes when we give the arginine, and then less frequently after that. We will ask to obtain a small amount of your urine now and whenever you need to pass urine. We will also ask you to breath into a tube every 30 minutes at first and later every few hours to check the amount of nitric oxide in your breath and how well your lungs work. These are tests used routinely in hospitals to check peoples’ lungs, and do not hurt.

We will also use an ultrasound test to measure the size of one of your blood vessels at the elbow plus blood flow within it. This technique is safe and painless, and does not cause any harm. The scan of your arm will be done with you resting, lying down and relaxing. We will then put a blood pressure cuff on your forearm and inflate the cuff for 4 minutes. When the cuff is let down, we will do a second scan. Inflating the cuff will be uncomfortable, like having your blood pressure recorded, but does not cause damage. Then there will be a break for 10-15 minutes. We will then do another scan with you resting and relaxing. We will repeat these ultrasound tests up to two more times, with the last time being tomorrow morning.

Your participation in the research will not cost you or your family any money. Your doctor may recommend some other tests and procedures to better diagnose and treat your infection. If you decide not to participate in the study, you will still receive this standard treatment and care for malaria. You may withdraw from the study at any time and still receive standard treatment and care. There will be no name or other ident-ification in any study report which will be published later on. You may contact Dr Rini on 901-301 827 at any time to answer any questions about the study. If you have any concerns about your rights or the conduct of the study you may contact Dr Liliana Kurniawan on 21-425 9860 or Linda Ward on 61-8-8922 7922.

***Benefits of the study:*** People who have a milder form of malaria like you usually get better quickly with the usual treatment for malaria and may not receive direct benefit from the extra arginine treatment study used in this study. However by understanding how much arginine we need to give to people with malaria and what effect it has in malaria, we hope to be able to give this extra treatment to people with bad malaria in future.

***Risks from the study:*** Arginine is a natural substance in the body and in many foods, but is also available as an injection in hospitals. It has been used as a standard drug in hospitals for many years in larger doses than we will use today. Even when used in bigger doses than we will use it is usually very safe, but you may have side effects if you receive arginine. Sometimes large doses can cause the blood pressure to go down and sometimes people may feel dizzy, feel sick, headache or flushing. Other times it can cause some of the salts in the blood to go up or down but it is very rare for this to cause problems. It can also cause irritation of the veins or very rarely an allergic reaction. Because we will use smaller doses, we do not expect these to be a problem for you. We will watch for any side-effects and can give you treatment if any of them develop.

**CONSENT TO PARTICIPATE IN A MALARIA RESEARCH STUDY**

**“Arginine in malaria: study 3”**

**This form means I can say “No”**

I have read and understood the information sheet attached, and have been given the opportunity to discuss it and ask questions. I agree that my samples can be used in the research outlined on the information sheet. I understand that I do not have to participate in this study. If I do not participate in the study I will still receive the usual treatment.

I also DO / DO NOT agree for my samples to be stored indefinitely for potential future use for research related to people respond to or are protected from malaria. I understand that my stored samples will not be used for any other purpose.

By my signature below I consent to participate:

___________________________ ______________________________ _____________

Name of study subject Signature of study subject/next of kin Date

_________________________________ ______________________________ _____________

Name of person obtaining consent Signature of person obtaining consent Date

**APPENDIX 1 List of Abbreviations**

ATS: American Thoracic Society

CAT 1: Cationic amino acid transporter 1

CAT 2: Cationic amino acid transporter 2

CK: Creatine Kinase

CL: Clearance

Cl^-^: Chloride

CM: Cerebral Malaria

DLCO: Lung diffusion for carbon monoxide, a measure of Gas Transfer

FMD: Flow mediated dilatation

GMP: Good Manufacturing Practice

GSH: Plasma glutathione

GSSG: Oxidised Glutathione

HCO3-: Bicarbonate

iSTAT: Brand of Portable Clinical Analyzer

K+: Potassium

Km: Equilibrium constant

LFTs: Liver Function Tests

MSHR: Menzies School of Health Research

NIHRD: National Institute for Health Research and Development

NONMEM: Computer software program to model non-linear pharmacokinetics of drugs.

NO: Nitric oxide

NOS1: Nitric oxide Synthase 1 or Neuronal Nitric oxide Synthase

NOS2: Nitric oxide Synthase 2 or Inducible Nitric oxide Synthase

NOS3: Nitric oxide Synthase 3 or Endothelial Nitric oxide Synthase

NOx: Nitric oxide metabolites, nitrate plus nitrite

PBMCs: Peripheral blood mononuclear cells

PK: Pharmacokinetic (change in arginine concentration over time)

PD: Pharmacodynamic (change in arginine effect over time)

RSMM: Rumah Sakit Mitra Masyarakat

sICAM-1: soluble intercellular adhesion molecule 1

SEAQUAMAT: South East Asian multi-center study of QUinine versus Artesunate for severe MAlaria Trial.

SM: Severe *Plasmodium falciparum* Malaria

SCD: Sickle Cell Disease

VOCS: Vaso-occlusive syndrome (found in SCD)

UF: Uncomplicated *Plasmodium falciparum* malaria

WHO: World Health Organization

Appendix 2

Table of Laboratory Tests to Be Done and Location Where Tests Will be Performed

| Type Of Specimen | Tests To Be Performed | Location of Tests |
| --- | --- | --- |
| Whole Blood | ISTAT EC8-(Glucose, Sodium, Potassium, Chloride, Urea, Hematocrit, Hemoglobin, PH, Base Excess, Anion Gap, Total CO_2_, PCO_2_) | RSMM-Timika |
| Whole Blood | Thick and Thin Films for Malaria parasites | RSMM-Timika |
| Whole Blood | Lactate, Phosphate | RSMM-Timika |
| Plasma | Liver function tests, Creatinine, creatine kinase | RSMM-Timika  Cross Checked in Darwin |
| Plasma | Arginine, other amino acids, NOx, ICAM-1 | MSHR, Darwin with participation of NIHRD scientist |
| Whole blood/Plasma | Markers of Oxidative Stress (incl. GSH:GSSG and cell superoxide release) | University of NSW, Sydney |
| Plasma | Endothelial microparticles | University of Marseille,  France |
| Urine | NO metabolites and Pterins | MSHR, Darwin |

In accordance with the 2003 MOU signed between Menzies School of Health Research and NIHRD, all specimens will be handled by the guidelines stipulated in that document. The attached “special agreement” sample MOU (with strict limitations on the use of samples) will be used when samples are sent to the lab of another organization (see appendix 5).

Appendix 3

Physiological Tests to be done and location of tests:

| Type of Specimen | Type of Tests | Location Of Tests |
| --- | --- | --- |
| Exhaled air | Exhaled Nitric Oxide using NIOX apparatus | RSMM-Timika |
| Exhaled air | Physiological Dead Space | RSMM-Timika |
| Exhaled air | Diffusing Capacity-DLCO to measure gas transfer in lungs | RSMM-Timika |
| Ultrasound imaging of forearm and finger | Endothelial Function Testing (NO-dependent) | RSMM-Timika |

Appendix 4

Standard Operating Procedure for Measuring Exhaled Nitric Oxide with the NIOX Analyzer in Accordance to ATS Guidelines

Steps

1. Explain to the patient the complete procedure in Bahasa Indonesian or English.
2. NIOX machine should already be turned on and running for greater than 4 hours.
3. Calibrate the NIOX if the last calibration was greater than 14 days.
4. Replace NIOX room air filter with a new filter for every patient. Filters are not to be reused.
5. Enter patient’s name, ID number, examiners name and any other comment in the NIOX
6. Select either the meter or balloon view
7. While seated or lying upright, patient exhales and then places NIOX filter in the mouth.
8. Inhale to total lung capacity over 2-3 seconds with NIOX filter in the mouth.
9. Patient exhales slowly and constantly keeping constant flow as measured by indicators on either the meter or balloon view.
10. Repeat until three valid measurements are obtained for analysis.

**Appendix 5: MOU for sample transfer**

See following MOU document


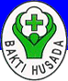


**NATIONAL INSTITUTE OF HEALTH RESEARCH & DEVELOPMENT**

**MENZIES SCHOOL OF HEALTH RESEARCH**

**HEALTH RESEARCH COLLABORATION**


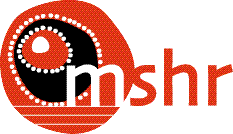


| National Institute of Health Research and Development (NIHRD)  Jl. Percetakan Negara No. 29  Jakarta 10560  Indonesia  PO Box 1226, Jakarta 10012  Telephone: 62-21-425 9860  Fax: 61-21-424 5386  Email: [selitbang@litbang.depkes.go.id](mailto:emilt@depkes.go.id) | International Health Program,  Menzies School of Health Research (MSHR)  PO Box 41096 Casuarina  Northern Territory 0811  Australia  Telephone: 61-8-8922 8932  Fax: 61-8-8927 5187  Email: international.health@menzies.edu.au |
| --- | --- |

MEMORANDUM OF UNDERSTANDING

Between

National Institute of Health Research and Development (NIHRD)-Menzies School of Health Research (MSHR)

And

…………(insert Organisation)…………………………………….

| Title of Project : |  |
| --- | --- |
| Agreement Date : |  |
| Agreement Expiry Date : |  |

Intended Purpose :

- (Samples) **will be sent from NIHRD-MSHR to** (Organisation) **for the purpose of** (outline the *specific* purpose for which samples are being transferred)**.**
- **The** (samples) **will be processed and destroyed/returned (delete one) to NIHRD-MSHR on completion of the research outlined above, and by the expiry date of, this Memorandum of Understanding.**
- **The** (samples) **will not be forwarded by** (Organisation) **to any third parties without the written agreement of the NIHRD-MSHR.**
- **Information gained, and by-products generated, from these samples will not be passed to any third party without the written agreement of the NIHRD-MSHR.**
- **The** (samples) **will not be used by** (Organisation) **for any purpose other than that outlined above.**

Ethics Approvals :

- **Where samples have been derived from human beings, the NIHRD and MSHR Human Research Ethics Committee (or similar authority) has approved the research proposed under this Agreement.**
- **No personal or other identifying information, will be disclosed to persons outside of the NIHRD-MSHR or otherwise published without the express consent of the individual/s or community/ies concerned.**

Intellectual Property and Outputs :

- **Analysis and publication of results will be jointly undertaken by NIHRD-MSHR and** (Organisation)**, to the satisfaction of both parties.**
- **All intellectual property arising from research undertaken on this collection will be jointly owned by the NIHRD-MSHR and** (Organisation), **to the satisfaction of all parties. NIHRD-MSHR will be informed of and involved in any development of intellectual property arising from this research.**
- **All individuals, from both NIHRD-MSHR and** (Organisation) **involved in the development of intellectual property arising from this research will be named authors on any patent application and the authors will benefit from commercialisation income distribution as per existing NIHRD and MSHR Intellectual Property Policies.**

Agreed Between :

|  |  |  |
| --- | --- | --- |
| **Senior NIHRD Researcher** | **Senior MSHR Researcher** | **(Other Organisation Senior Researcher)** |
| **(Position Title)** | **(Position Title)** | **(Position Title)** |
| **(Date)** | **(Date)** | **(Date)** |
